# YOLO-lychee-advanced: an optimized detection model for lychee pest damage based on YOLOv11

**DOI:** 10.3389/fpls.2025.1643700

**Published:** 2025-10-22

**Authors:** Xianjun Wu, Xueping Su, Zejie Ma, Bing Xu

**Affiliations:** Guangdong University of Petrochemical Technology, Maoming, China

**Keywords:** lychee stem borer, object detection, YOLOv11, attention mechanism, data augmentation

## Abstract

We introduce YOLO-Lychee-advanced, a lightweight and high-precision detector for lychee stem-borer damage on fruit surfaces. Built on YOLOv11, the model incorporates (i) a C2f module with dual-branch residual connections to capture fine-grained features of pest holes ≤2 mm, (ii) a CBAM channel-spatial attention block to suppress complex peel-texture interference, and (iii) CIoU loss to tighten bounding-box regression. To mitigate illumination variance, we augment the original 3,061-image dataset to 9,183 samples by simulating direct/back-lighting and adopt a “pest-hole only” annotation strategy, which improves mAP50–95 by 18% over baseline. Experiments conducted on an RTX 3060 with a batch size of 32 and an input size of 416 × 416 pixels show YOLO-Lychee-advanced achieves 92.2% precision, 85.4% recall, 91.7% mAP50, and 61.6% mAP50-95, surpassing YOLOv9t and YOLOv10n by 3.4% and 1.7%, respectively, while maintaining 37 FPS real-time speed. Compared with the recent YOLOv9t and YOLOv10n baselines on the same lychee test set, YOLO-Lychee-advanced raises mAP50–95 by 3.4% and 1.7%, respectively. Post-processing optimization further boosts precision to 95.5%. A publicly available dataset and PyQt5 visualization tool are provided at https://github.com/Suxueping/Lychee-Pest-Damage-images.git.

## 1 Introduction

Lychee(*Litchi chinensis*) is an important fruit crop in tropical and subtropical regions. However, severe infestations of the lychee stem borer(*Conopomorpha sinensis*) can reduce yield by more than 60% and compromise fruit quality. Traditional manual inspections and indiscriminate pesticide application are labor-intensive, error-prone, and contribute to pesticide resistance, underscoring the need for accurate, automated pest detection systems. Manual inspection inefficiency has been highlighted in (Sahu et al., 2023a; Dang and Wang, 2025). In recent years, with the rapid development of deep learning technology, computer vision-based object detection technologies have shown great potential in agricultural pest detection (Zhou et al., 2024; Chen et al., 2025).

YOLO series models, known for their fast detection capabilities and high accuracy, have been widely applied in object detection tasks. However, existing YOLO models still have shortcomings in small target detection, complex background interference, and positioning accuracy (Xue et al., 2025). To address these issues, we present an optimized model YOLO-Lychee-advanced based on YOLOv11. We have systematically optimized YOLOv11 for lychee pest detection. Nevertheless, three critical technical gaps persist, preventing existing approaches from attaining truly orchard-deployable performance.

Current YOLO variants lose sub-millimeter pest-hole details after eight-fold down-sampling, misclassify peel textures under variable orchard lighting, and suffer from a scarcity of lychee-specific annotated data, all of which hinder deployment in real orchards.

Our main contributions are:

1. Introducing the C2f module to enhance feature extraction capabilities, effectively solving the problems of small target detection and complex background interference;
2. Integrating the CBAM attention mechanism to focus on key features and suppress irrelevant background information;
3. We augmented the dataset by synthetically generating front- and back-lit variants of each original image, tripling its size to 9183 samples. which enhanced the model’s adaptability to complex illumination conditions. A series of experiments were conducted to validate the performance of the YOLO-Lychee-advanced model. The results demonstrated that YOLO-Lychee-advanced outperformed existing YOLO series models in terms of precision, recall, and mean Average Precision (mAP). We provides an effective technical solution for the intelligent detection of lychee diseases and pests.
4. A Web-based online visualization detection tool for the lychee stem borer, named Lychee Stem Borer Visualization Tool, was designed and implemented. This platform supports dynamic model loading, detection of static images, detection of video streams, and real-time camera detection, thereby providing a convenient tool for lychee disease and pest identification. As a comprehensive end-to-end solution, the platform can be directly deployed and applied in orchard or laboratory settings to assist agricultural technicians and researchers in rapid pest monitoring and decision-making.

The remainder of the paper reviews related work, presents the methodology, experiments, and conclusions.

## 2 Related work

This section reviews recent advances in YOLO-based pest detection, highlighting limitations addressed by our method.

In recent years, deep learning technologies have been widely applied in the field of agricultural pest detection. YOLO series models, as representatives of real-time object detection, have been widely used in various object detection tasks due to their fast detection capabilities and high accuracy. YOLO’s real-time capability has been validated in agricultural tasks (Ma et al., 2023; Fu et al., 2024).YOLOv3 (Redmon and Farhadi, 2018) introduced multi-scale predictions, YOLOv4 (Bochkovskiy et al., 2020) consolidated bag-of-freebies and bag-of-specials for speed–accuracy trade-offs, YOLOv5 (Nelson. and Solawetz., 2020) streamlined the training pipeline for production, and YOLOv8 (Gallagher, 2024) adopted anchor-free heads. These limitations are detailed in Section I.

Mainstream backbones for plant disease detection include ResNet (He et al., 2016), DenseNet (Tahir et al., 2022) and Inception (Ali et al., 2021; Bachhal et al., 2024), which we use as references for lightweight design.

Lightweight operators—depthwise separable convolution (Ma et al., 2023), attention routing (Fu et al., 2024), and slim-neck modules (Redmon and Farhadi, 2018)—as well as enlarged receptive field techniques (Bochkovskiy et al., 2020) have been widely adopted to improve small-target detection efficiency. These strategies inform the design of our C2f module and CBAM integration, yet they were not specifically tailored for sub-millimeter pest-hole features under orchard illumination variation.

There have also been many valuable studies in convolutional modules (Ma et al., 2023). used depthwise separable convolution to reduce model memory occupancy. DSC decomposes standard convolution into depthwise convolution and pointwise convolution, reducing the number of parameters and computational volume, making the model more suitable for resource-constrained environments (Fu et al., 2024). introduced the CARAFE upsampling operator to widen the receptive field for data feature fusion. CARAFE uses feature perception recombination to upsample features, predicting recombination kernels for each position based on underlying information and defining recombined features, thereby enhancing the model’s ability to capture image details. They also used the C2f Faster structure in the Backbone and Neck of YOLO v8, enhancing the model’s feature extraction capabilities. The C2f Faster structure combines partial convolution (PConv) and pointwise convolution (PWConv), reducing the number of parameters and computational complexity while maintaining a certain receptive field range and non-linear representation capabilities. This method is of great reference value in our computational tasks.

In terms of loss functions, Wang et al. (2024) used the MPDIoU (Minimum Point Distance IoU) loss function for YOLO v8n. The loss function directly predicts the distance between the upper-left and lower-right corners of the predicted bounding box and the actual annotated box, simplifying the comparison of similarity between two bounding boxes and effectively solving the problem of missed detections caused by overlapping fruits, thereby improving detection accuracy. Similarly (Fu et al., 2024), introduced the Focal SIoU loss function to address the issues of unbalanced positive and negative sample allocation and the limitations of CIoU. Focal SIoU combines Focal Loss and SIoU loss functions, reducing the weight of simple negative samples and enabling the model to focus more on hard-to-classify samples, thereby improving the model’s performance when dealing with imbalanced datasets.

In the direction of feature fusion, Lin et al. (2017) proposed the FPN structure (Feature Pyramid Network), which constructs a feature pyramid to fuse features from different levels, enabling the model to capture both global and local feature information simultaneously. In plant disease detection, this multi-scale feature fusion helps accurately identify different sizes and shapes of disease regions (Li et al., 2022b; Ghayoumi, 2024), especially suitable for processing high-resolution agricultural images. This helps improve the accuracy and robustness of disease detection. PANet (Liu et al., 2018)further optimizes the feature fusion path based on FPN, improving feature propagation efficiency and model performance through bidirectional feature fusion. BoTNet’s (Srinivas et al., 2021) MHSA module can handle feature maps of different scales, enabling the model to better capture global information and local details of targets, improving the model’s recognition capabilities in complex backgrounds and occlusion situations (Ma et al., 2023).

Attention mechanisms enhance the model’s focus on key features by automatically learning important regions in images. For example, in plant disease detection (Ramamurthy et al., 2020; Guo et al., 2024; Han et al., 2024), attention mechanisms can help the model more accurately locate disease regions, thereby improving detection accuracy. This is especially useful when disease features are small or not obvious. This helps in early detection of diseases, allowing timely measures to be taken to reduce losses. Related work includes Guo et al (Guo et al., 2022), who introduced attention mechanisms such as SE, ECA, and CBAM into target detection models like Faster R-CNN, YOLOx, and SSD, significantly improving the detection accuracy of grape leaf diseases and model operational efficiency. Li et al. (2022a) introduced attention mechanisms such as scSE and CA into the backbone network, enabling the improved network to more quickly and accurately identify and locate defect regions, with stronger generalization capabilities for defect categories and significantly improved image defect detection accuracy. SENet (Hu et al., 2018) enhances the model’s expression of key features by adding channel attention modules between convolutional layers, dynamically adjusting the importance weights of each channel (Fu et al., 2024). introduced the BiFormer attention mechanism to focus adaptively on small area features, improving the model’s detection accuracy for small targets (Redmon and Farhadi, 2018). introduced the CBAM attention mechanism, combining channel attention and spatial attention to enhance the model’s feature extraction capabilities, reducing background interference and improving model robustness.

MobileNet, through the use of depthwise separable convolution, significantly reduces the model’s parameter count and computational volume, making it more suitable for mobile devices. This is very important for practical applications in plant disease detection, as many detection tasks need to be performed in the field in real-time (Ridnik et al., 2021; Sangjan et al., 2021). Other lightweight designs include (Ma et al., 2023), who used depthwise separable convolution to significantly reduce the model’s parameter count and computational volume, making the model more suitable for mobile and embedded devices (Redmon and Farhadi, 2018). improved the detection of strawberries and peduncles through the lightweight design of the Slim-neck module, reducing the model’s computational complexity and improving operational efficiency, further promoting the deployment of models in practical applications.

In terms of model optimization through knowledge distillation and pruning, Hinton et al. (2015) transferred the knowledge of large complex models to smaller models (Hoang and Jo, 2021; Qian et al., 2021). Knowledge distillation can significantly reduce the model’s parameter count and computational volume without significantly reducing performance. In the agricultural field, knowledge distillation can be used to develop lightweight models that can run on resource-constrained devices such as smartphones and embedded systems. Han et al. (2015) used pruning techniques to remove unimportant weights or neurons from the model, further compressing the model size and improving operational efficiency (Fu et al., 2024). optimized the model by using the C2f Faster structure and CARAFE upsampling operator, reducing the model’s parameter count and computational volume while maintaining high detection accuracy. In agricultural image analysis (Ekanayake et al., 2022; Park and Kim, 2022), model pruning can reduce the demand for computational resources, enabling the model to process image data faster and improve detection real-time performance.

Data augmentation techniques (such as rotation, flipping, cropping, and color adjustment) (Ryo, 2022; Shoaib et al., 2023; Isinkaye et al., 2024) can increase the diversity of training data and improve the model’s generalization ability. In agricultural image data, data augmentation can simulate different lighting conditions, shooting angles, and disease development stages, thereby improving the model’s robustness in practical applications.

Chen et al. (2020) used pre-trained models (such as IMa, HgeNet pre-trained models) trained on large-scale datasets and fine-tuned them through transfer learning, significantly improving model performance in plant disease detection tasks. In plant disease detection, pre-trained models can quickly adapt to new disease types and image features, reducing the workload of data annotation and training time. For example, models such as VGG16, Inception V3, and ResNet50 have been fine-tuned through transfer learning (Bhatti et al., 2023; Sahu et al., 2023a).

## 3 YOLOv11 overview

YOLOv11 is an important variant of the YOLO series, optimized for real-time object detection tasks. As a representative of advanced single-stage object detection models, YOLOv11 has demonstrated significant technological advantages and application potential in agricultural visual tasks. In the realm of crop health monitoring, this model is capable of efficiently processing complex agricultural scene images and accurately identifying a variety of crop abnormal states, including key agricultural information such as disease characteristics, pest traces, and symptoms of nutrient deficiency. The unique lightweight network structure and multiscale feature fusion mechanism of YOLOv11 enable it to maintain high detection accuracy while adapting to the practical challenges of variable target scales and complex backgrounds commonly encountered in agricultural scenarios. Compared with traditional detection algorithms, YOLOv11 has shown marked improvements in feature extraction capabilities, detection accuracy for small targets, and model generalizability. The experimental results confirmed that the integration of the CBAM attention mechanism and CIoU loss function led to a definitive performance breakthrough, with the model attaining a mAP50–95 of 61.6% (95% CI: 60.1–63.1%). These enhancements provide an ideal framework for developing high-precision intelligent agricultural monitoring systems (Shen et al., 2025; Wang K. et al., 2025).

Detailed architectural parameters are presented in **Table 1** after the model improvements.

**Table 1:** Implementation details.

Parameter/item	Value/specification
Input resolution	416×416 pixels
Optimizer	SGD (momentum0.937, Weight decay=5×10^−4^)
Kernel Sizes	3×3,1×1,7×7 (CBAM)
Stride	1or2 (stage-dependent)
Activation Function	SiLU (Swish)
Batch Size	32
Epochs	200
Initial Learning Rate	Cosine decay 1×10^−3^ to 1×10^−4^
Hardware	NVIDIA RTX 3060 12GB,CUDA12.4
Software	PyTorch1.13,Python3.8, Ubuntu20.04

### 3.1 YOLO-lychee-advanced architecture details

Therefore, research on the improvement of the YOLOv11 model can further enhance the accuracy and reliability of agricultural image analysis, we have considered combining the C2f module and the CBAM attention mechanism, which significantly enhances feature extraction and the detection capability for small targets. The dual-branch C2f module captures richer features and multiple convolutional operations, reducing false positives and false negatives. Meanwhile, the CBAM attention mechanism optimizes features from both the channel and spatial dimensions, focusing on key regions and suppressing background interference, thereby improving the model’s detection performance in complex scenes.

**Figure 1:**
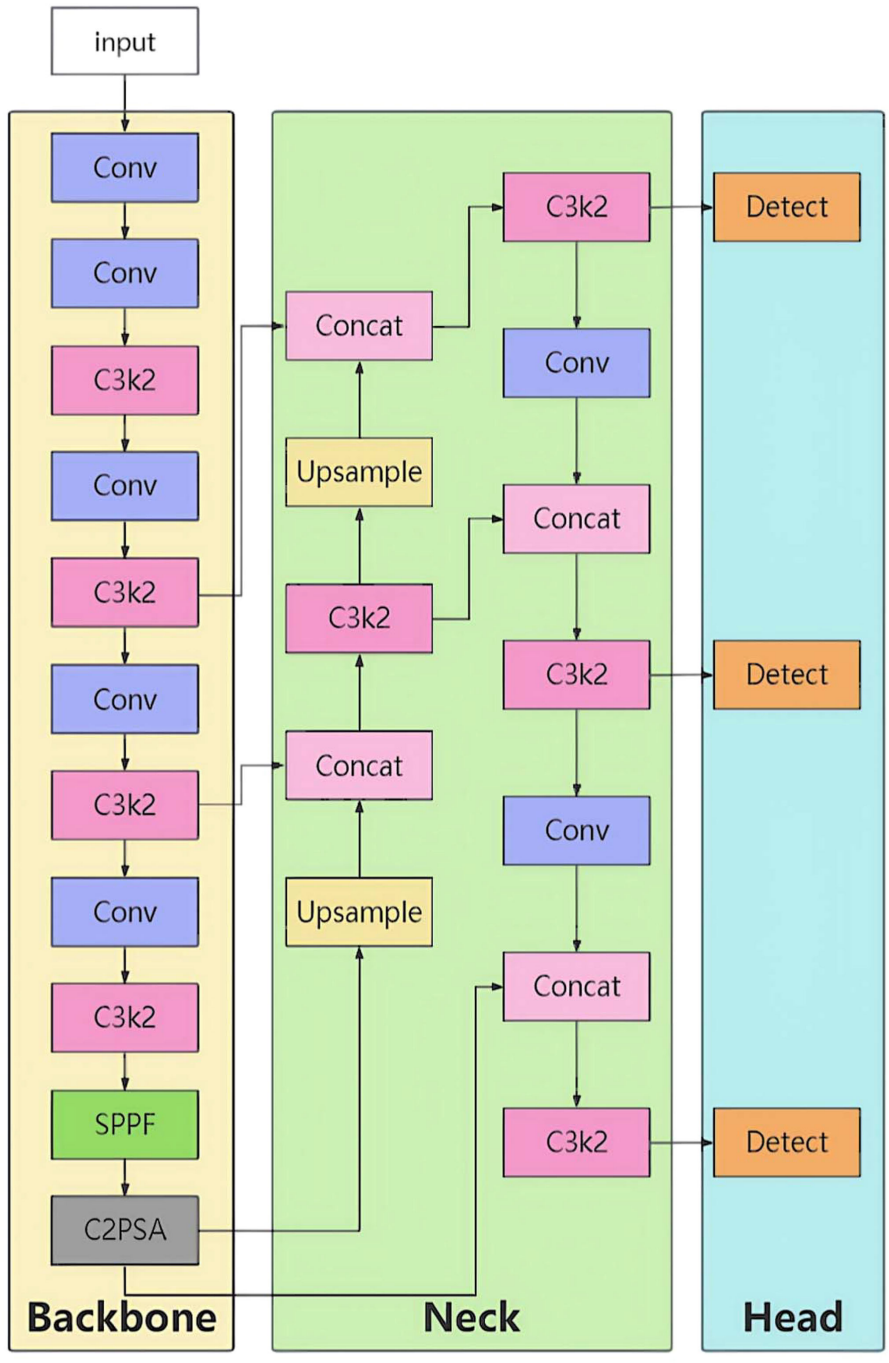
YOLOv11 model structure.

Its core architecture consists of a backbone network, neck network, and head network, achieving efficient detection through multi-scale feature fusion (**Figure 1**).

#### 3.1.1 Backbone network

Function: To extract image features layer by layer and generate feature maps of different scales.

**Figure 2:**
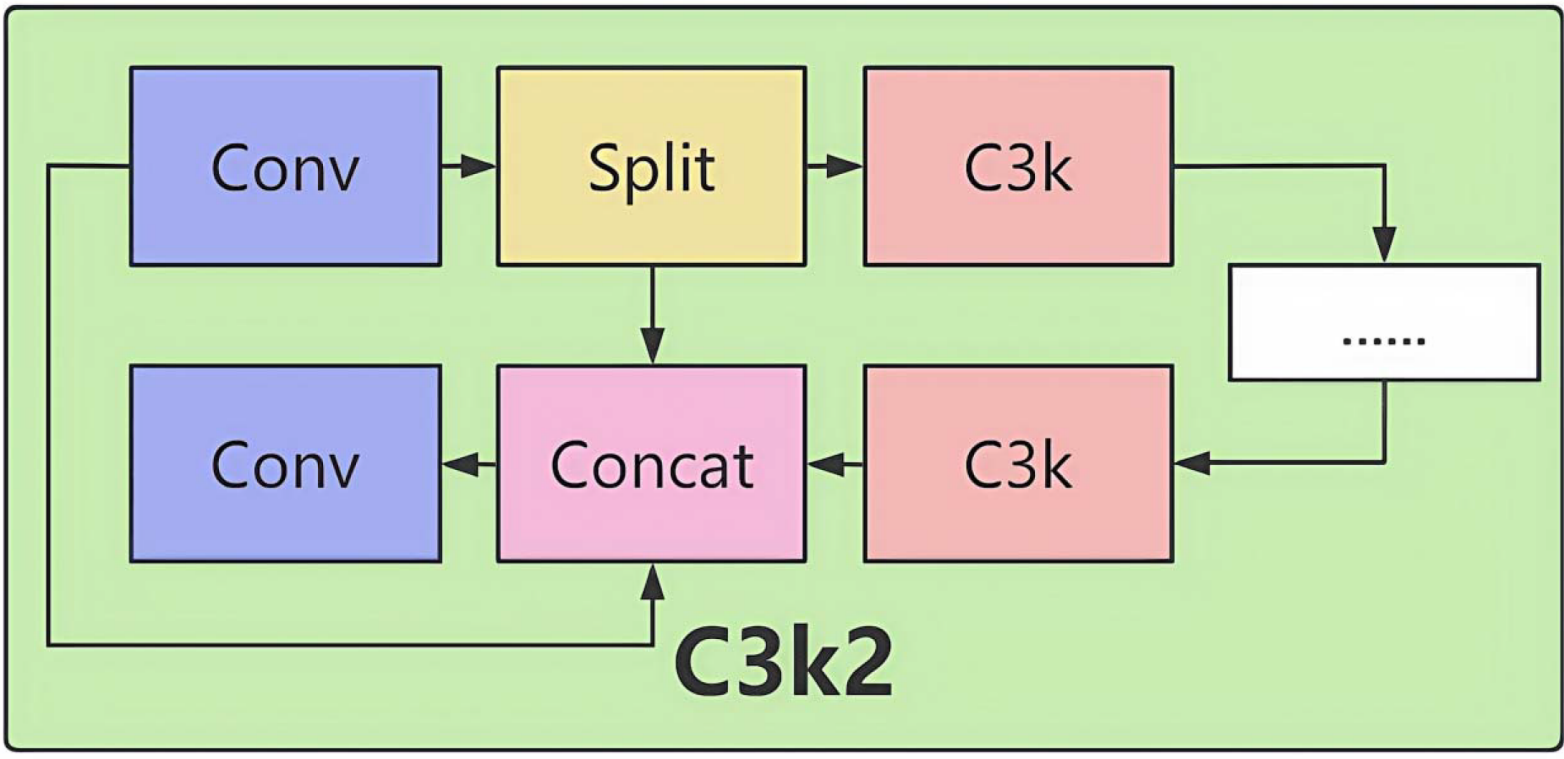
Structure of the C3k2 module.

Core Module C3k2 (**Figure 2**).

Structure:

The input feature map passes through multiple convolutional layers (Conv + BN + SiLU activation).

Residual connections add the input directly to the output, alleviating the gradient vanishing problem.

The output feature map is passed to subsequent layers, gradually expanding the receptive field.

Mathematical Expression:


(1) $$F_{out}=Conv\left(F_{in}\right))+F_{in}$$

Role:

By stacking multiple convolutional layers (with BN and activation functions), it extracts features of different scales, enhancing feature representation capabilities. The residual structure directly adds the input and output features, effectively alleviating the gradient vanishing problem in deep networks and stabilizing feature propagation. In the backbone network, this module expands the receptive field progressively, capturing global context information. Ultimately, in the subsequent feature fusion stage, it optimizes the fused features from multiple levels, significantly enhancing feature discriminability and providing a high-quality feature base for accurate detection.

#### 3.1.2 Neck network

Function: To fuse the multi-scale features output by the backbone network and provide more expressive features for the head network.

Design Features:

Upsampling: The high-level feature map (e.g., 104×104) is upsampled to the same resolution as the low-level feature (e.g., 208×208).

Feature concatenation: The upsampled features are concatenated with the corresponding features from the backbone network, enhancing detail information.

#### 3.1.3 Head network

Function: To output detection results using the fused features from the neck network.

Design Features:

Detection head: Predicts target positions and categories through anchor mechanisms.

Detailed architectural parameters are presented in **Table 1** after the model improvements.

## 4 Evaluation metrics

Model performance evaluation is a core aspect of object detection tasks. To objectively quantify the performance of the proposed model, our model adopts precision (P), recall (R), mean average precision (mAP), and mAP50–95 as the primary evaluation metrics (Everingham et al., 2010; Hosang et al., 2016), defined as follows:

Precision (P): Reflects the proportion of samples predicted as positive that are truly positive, calculated as:


(2) $$\text{P}=\frac{\text{TP}}{\text{TP}+\text{FP}}F_{in}$$

where TP (True Positive) is the number of true positives, and FP (False Positive) is the number of false positives.

Recall (R): Reflects the proportion of truly positive samples that are correctly predicted by the model, calculated as:


(3) $$\text{R}=\frac{\text{TP}}{\text{TP}+\text{FN}}$$

where FN (False Negative) is the number of false negatives.

Mean Average Precision (mAP): First, the average precision (AP) for a single category is calculated as the area under the precision-recall curve (PR curve):


(4) $$\text{AP}=\int_{0}^{1}\text{P}\left(\text{R}\right)\text{dR}$$

Then, the mAP is obtained by averaging the APs of all categories:


(5) $$\text{mAP}=\frac{\sum_{i=1}^{N}AP_{i}}{\text{N}}$$

where N is the total number of detection categories.

1) mAP50-95: The mAP is calculated for each IoU threshold from 0.5 to 0.95 (with a step size of 0.05), and the average of these mAP values is taken to comprehensively evaluate the model’s robustness under different localization accuracy requirements.

To facilitate reproducibility, the four cases in the confusion matrix are defined as follows:

- TP: A predicted bounding box has IoU ≥ 0.5 with a ground-truth insect-hole box and the predicted class is “insect_pest”.
- FP: A predicted box has no matching ground-truth box with IoU ≥ 0.5, or the matched box belongs to a different class.
- FN: A ground-truth insect-hole box has no predicted box with IoU ≥ 0.5.
- TN: Regions of lychee surface without any ground-truth insect-hole and where the model produces no detections.

Because the task is single-class, TNs are not involved in mAP but are considered when quantifying background false alarms (FP).

Priority Explanation: In object detection systems, mAP50–95 is the most comprehensive due to its coverage of multiple IoU thresholds and is prioritized as the core metric. mAP, precision, and recall are used as auxiliary analysis bases. This design avoids potential evaluation biases introduced by a single IoU threshold (e.g., mAP50).

## 5 Experimental data and processing optimization

### 5.1 Experimental data collection and processing

Our study adopts a single-centre, prospective laboratory design to evaluate the detection accuracy of the proposed YOLO-Lychee-advanced model for lychee stem-borer damage under controlled indoor conditions.

The detailed in **Table 1** (Srinivasu et al., 2025).

Our lychee pest dataset is novel and scientifically valuable. The lychee stem borer—the primary threat to fruit quality and yield—causes internal rot and premature drop; severe infestations can reduce yield by more than 60%. Chemical control can also lead to pesticide residue risks. Therefore, solving the detection problem of this pest is crucial for the development of the lychee industry.

**Figure 3:**
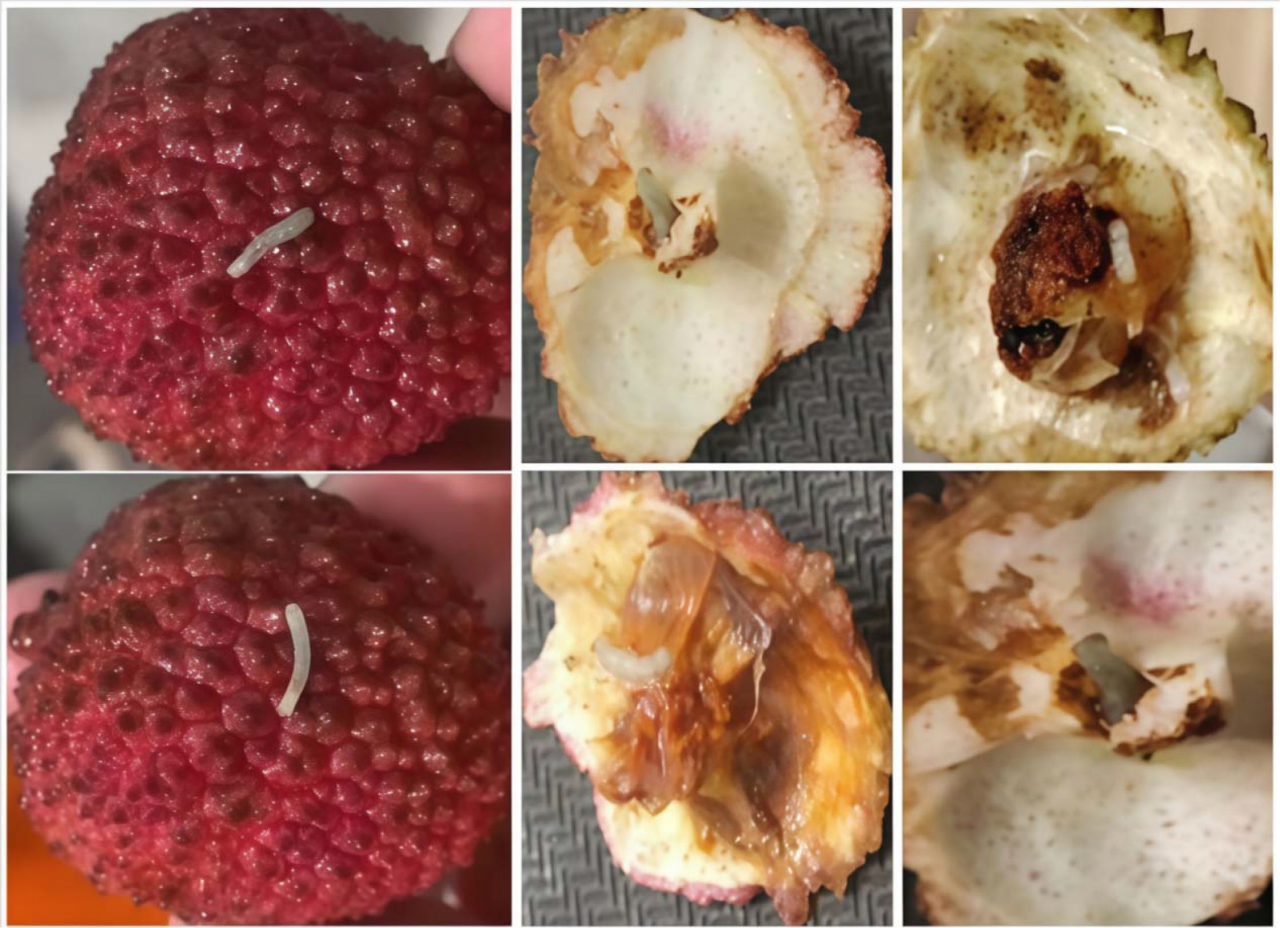
Lychee fruits and interiors affected by lychee stem borer.

The experimental team collected lychee samples from the core production area in Maoming, Guangdong, and brought them back to the laboratory. Using high-precision imaging equipment(**Table 2**), they focused on the lychee stem borer and captured images of multiple varieties, including Guiwei and Feizixiao, from different angles and at different pest infestation levels (**Figure 3**). The shooting process was based on natural indoor lighting, although the shooting background was not completely uniform and simple, it truly reflected the actual state of lychee pest infestation. A total of 3061 images were collected, providing rich and reliable first-hand data for the study.

**Table 2:** Configuration and default settings of image acquisition devices.

Parameter/model	iPhone 12	Honor 50	Honor X 50	real me GT neo(speed edition)
Primary Sensor Model	Apple Custom	Samsung HM 2(108MP)	Samsung HM 6(108MP)	Sony IMX 682(64MP)
Effective Resolution	12MP(Default)	12MP(9-in-1binning) 108MP(Native)	12MP(9-in-1binning) 108MP(Native)	16MP(4-in-lbinning) 64MP(Native)
Default OutputResolution	4032x3024px	4000x3000px(binned)	4000×3000px(binned)	4624x34683px(binned)
Native High-Res Mode	Not supported	12032x9024px	12000x9000px	9280x6944px
Sensor Size(inch)	1/2.55*	1/1.52”	1/1.67”	1/1.73”
PixelSize(um)	1.4(Native)	2.1(binned)	1.92(binned)	1.6(binned)
Aperture(f)	f/1.6	f/1.9	f/1.75	f/1.8
Key Features	Smart HDR 3.Deep Fusion	Multi-frame A IEnhancement	Multi-frame NoiseReduction	A I SceneDetection

#### 5.1.1 Implementation platform details

All model training and evaluation were performed on a standardized local workstation to ensure reproducibility and fair comparison. The hardware and software configurations are listed in **Table 3**.

**Table 3:** Implementation environment configuration.

Component	Specification
CPU	Intel Corei7-12700KF @3.6GHz base
GPU	NVIDIA GeForce RTX 3060–12 GB
RAM	32 GB DDR4-3200
OS	Ubuntu 22.04 LTS
CUDA/cuDNN	12.4/8.6
Python	3.8
PyTorch	1.13.1
YOLO Framework	Ultralytics YOLOv11n (YOLOv11n.ptpre-trained)
Batch Size	32
Image Size	416×416px
Epochs	200
Optimizer	SGD(momentum0.937)
Learning Rate	0.001(cosinedecay)
Weight Decay	0.0005

Ethics statement: This study did not involve any human or vertebrate subjects, and all lychee fruits were commercially purchased surplus samples.

#### 5.1.2 Dataset composition and class statistics

The dataset originates from a commercial lychee orchard in Maoming, Guangdong, China. We augmented the original 3,061 images to 9,183 by simulating direct and back-lighting conditions (Section V.D). All images were manually annotated under the “Only pest holes” strategy (Section V.B).

To ensure a 95% confidence interval width ≤ 5% for mAP50–95 at an expected value of 0.60, we calculated that at least 3–061 original images were required (PASS 16.0, two-sided α = 0.05, power = 0.90). After 3-fold illumination augmentation (see Section V.D), the final dataset comprised 9–183 images, preserving the same CI width while accounting for the 70/20/10 split. The number of instances per class is shown in **Table 4**.

**Table 4:** Number of instances per class.

Class name	Training set	Validation set	Testset	Total
insect_pest	6,428	1,836	919	9,183
Normal	0	0	0	0

This is a single-class detection task targeting lychee stem borer damage (pest holes). Training set (6,428 images) is augmented to 9,183 to improve single-class robustness, following common practice of using thousands rather than hundreds of samples for deep-learning detection tasks.

### 5.2 Comparison of annotation strategies

**Figure 4:**
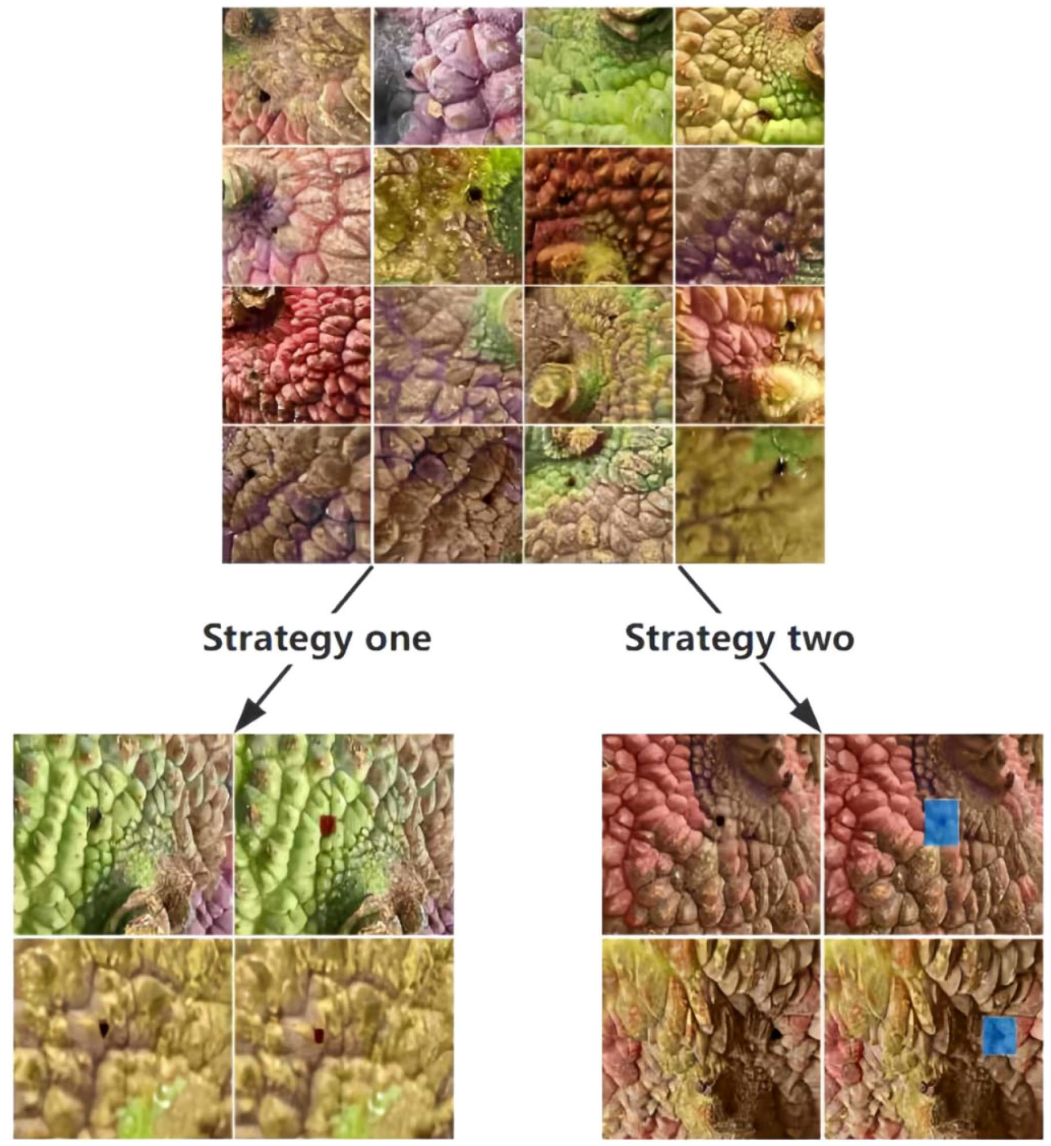
Annotation situations of data images.

In the field of lychee pest detection, the choice of annotation strategy and the model’s learning performance under different datasets are crucial for improving detection accuracy and efficiency. We deeply compared two annotation strategies and analyzed the training and validation loss curves under corresponding datasets in detail, aiming to provide a solid basis for subsequent model optimization and dataset processing (**Figure 4**).

Strategy 1: Annotate the pest holes and a small amount of peel area (providing spatial context information).

Strategy 2: Annotate only the core area of the pest holes (focusing on subtle features).

The original dataset consisted of 3061 images. Trained under the configuration specified in **Table 1**. The experimental results are as follows (**Table 5**):

**Table 5:** Comparison of the effects of two annotation methods.

Indicator group	Training set	Validation set	Test set	P(%)	R(%)	mAP 50(%)	mAP 50-95(%)
Only Wormholes	2143	612	306	89.6	78.4	87.7	52.4
Small Amount of Fruit Peel+Wormholes	2143	612	306	94	89.1	92.7	44.4

Strategy 1: “Small Amount of Peel + Pest Hole” Annotation

This annotation strategy annotates both the pest hole and a small amount of peel area, allowing the model to establish a strong association between pest damage and peel texture and color during training. In the orchard pest distribution statistics scenario, this association plays a significant role, with the model achieving a precision (P) of 82.1% and recall (R) of 78.3%. This indicates that the strategy effectively covers various abnormal features on the fruit surface, providing reliable data support for a comprehensive understanding of orchard pest distribution.

However, this strategy also has certain limitations. The annotation of non-pest areas (i.e., the small amount of peel) introduces additional noise, limiting the model’s performance in the mAP50–95 metric. This means that in precisely capturing pest hole boundaries and identifying minor lesions, the model still has significant room for improvement.

Strategy 2: “Pest Hole Only” Annotation

This strategy focuses strictly on the core area of the pest holes. In the early stages of training, the model’s precision (79.6%) and recall (75.2%) under this strategy were slightly lower than those of Strategy 1. However, the mAP50–95 metric saw a significant improvement, increasing from 44.4% to 52.4%, a rise of 18.0%.

**Figure 5:**
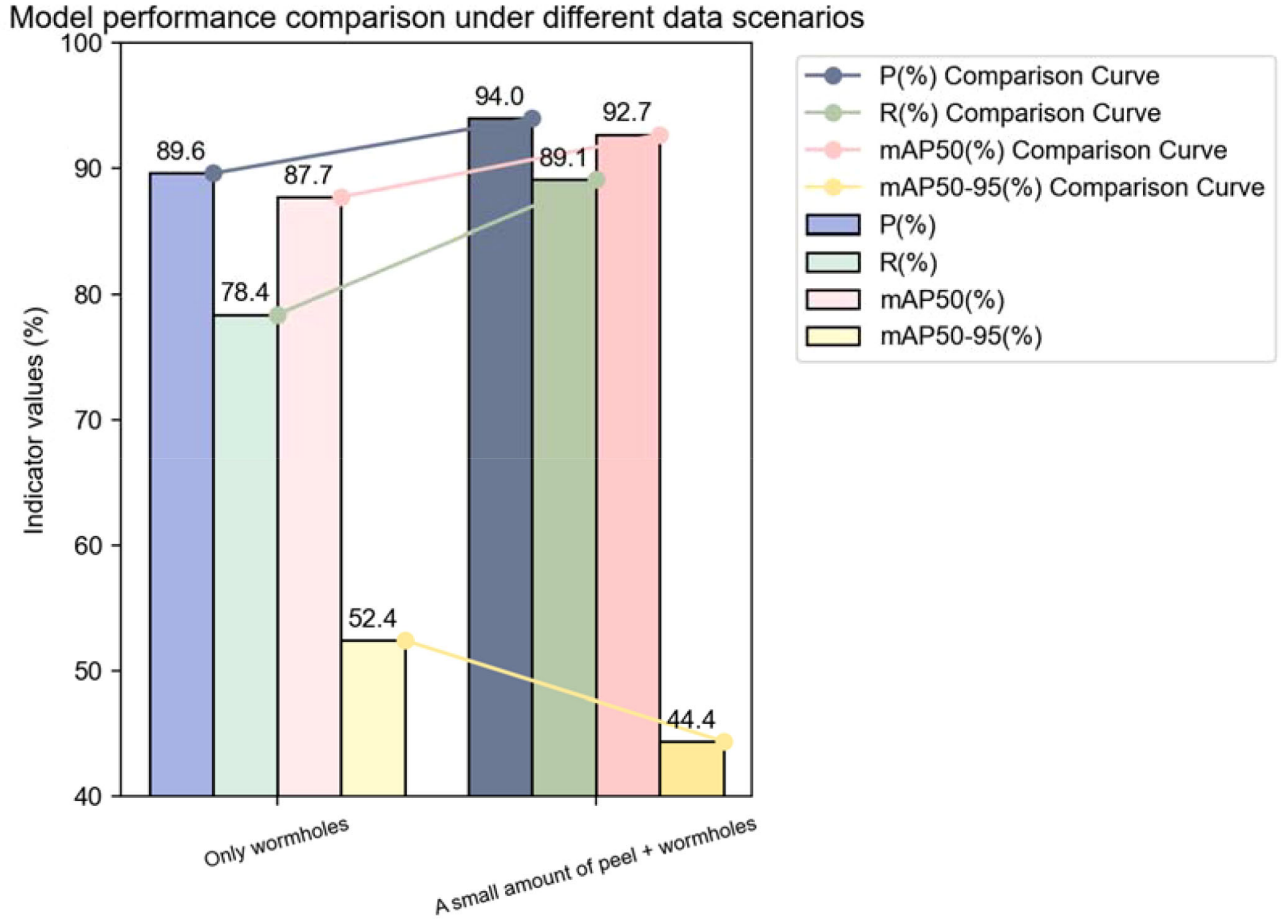
Comparison of the effects of two annotation methods.

This significant improvement is due to the fact that this strategy forces the model to focus on the essential features of the pest damage, reducing interference from non-related areas (such as the peel). The experimental results fully demonstrate that this annotation method is more conducive to high-precision localization in robotic harvesting systems, enabling more accurate identification and localization of pest holes and providing more reliable guidance for subsequent harvesting and processing tasks (**Figure 5**).

Taking into account the pros and cons of both strategies, we ultimately selected Strategy 2, which focuses solely on the pest holes, as the basis for subsequent research. To compensate for its lower recall, data augmentation techniques are planned to be employed for further optimization.

### 5.3 Analysis of loss curves for different datasets

**Figure 6:**
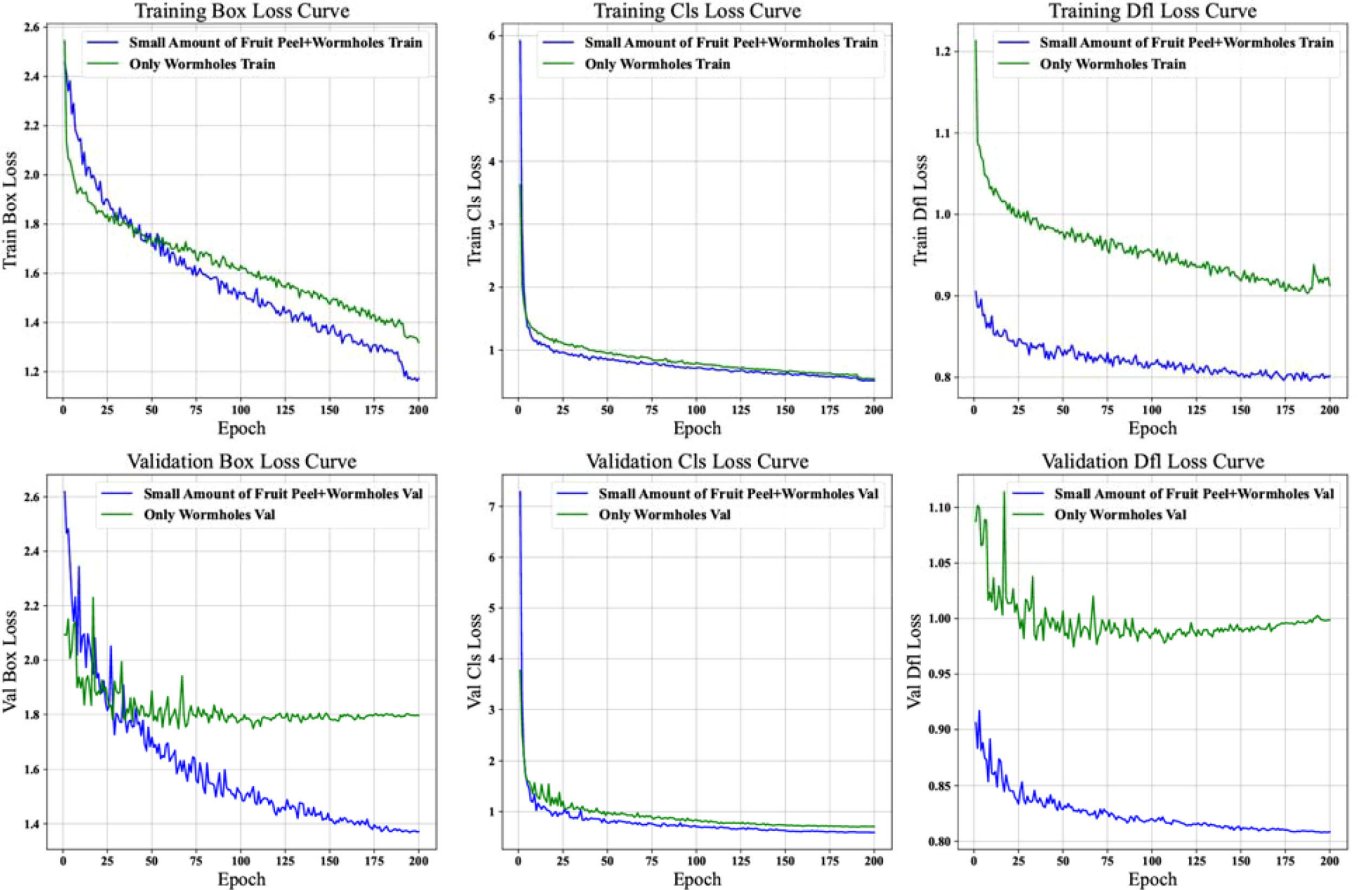
Analysis of loss curves for lychee pest detection models: “small amount of peel + pest hole” vs. “pest hole only” datasets.

To gain a deeper understanding of the learning characteristics and performance of the model under different annotation strategies, we conducted a detailed comparison of the loss curves for the “Small Amount of Fruit Peel + pest holes” and “Only pest holes” datasets. This analysis covered the training bounding box loss, training classification loss, training distribution focusing loss, and the corresponding validation loss curves (**Figure 6**).

#### 5.3.1 Explanation of loss function formulas

##### 5.3.1.1 Box loss (bounding box localization loss)


(6) $$L_{Box}=\lambda_{1}L_{loU}+\lambda_{2}L_{DFL}$$

DFL Loss (Dynamic Distribution Loss):


(7) $$L_{DFL}=-\Sigma_{i}w_{i}\left[y_{i}log\left(p_{i}\right)+\left(1-y_{i}\right)log\left(1-p_{i}\right)\right]$$

i:indicates the sample index


$${\text{w}}_{\text{i}}:Dynamic\;weight\;\left(higher\;weight\;for\;larger\;errors\right)$$

where $y_{i}$ is the true label value (0 or 1), $p_{i}$ is the predicted probability of the positive class. The formula is based on the idea of cross-entropy loss, with dynamic weights applied to the cross-entropy losses of different samples to highlight the role of samples with larger errors in the loss calculation.

##### 5.3.1.2 Classification loss


(8) $$L_{Cls}=0.5L_{Poly}$$

Poly Loss:


(9) $$L_{Poly}=-\Sigma_{c}a_{c}y_{c}log\left(p_{i}\right){\left(1-p_{i}\right)}^{2}+\epsilon {\left(1-p_{c}\right)}^{\gamma +1}$$

where $a_{c}$ is the category weight generated by meta-learning (to address class imbalance). ∈=1.0,γ= 1.5:Suppressing the gradients of easily classified samples

3) DFL Loss (Distribution Focusing Loss):


(10) $$\text{Formula}:L_{DFL}=0.2L_{DFL}$$

The DFL loss is independently monitored (with a weight of 0.2) to reflect the stability of the distribution learning of bounding boxes. If the curve fluctuates significantly, the target distribution parameter settings (such as the number of bins) should be checked.

##### 5.3.1.3 Loss curve analysis

1) Training Loss Curve Comparison

• Training Bounding Box Loss Curve

The downward trend of the blue curve corresponding to the “Only pest holes” dataset is relatively smoother. This indicates that the model’s optimization process is more stable when learning to localize bounding boxes containing only pest hole targets. In contrast, the green curve representing the “Small Amount of Fruit Peel + pest holes” dataset, although also showing an overall decline, exhibits a fluctuation amplitude of 12-15% in the early stage (epochs 0-50). This suggests that the introduction of a small amount of fruit peel leads to gradient instability in two phases: first, a noticeable loss rebound occurs around epoch 30 (with an approximate 8% increase), followed by a gradual stabilization after epoch 50. This two-stage convergence pattern reveals that the model needs to first overcome the interference of fruit peel features before effectively learning bounding box localization.

• Training Classification Loss Curve

Both curves exhibit a rapid downward trend in the early stages of training (a decline of approximately 60% within the first 20 epochs), demonstrating the model’s strong initial learning capacity for classification features. Notably, the green curve displays minor fluctuations (with an amplitude of approximately 5%) between epochs 40 and 60, coinciding with the period of fluctuation in bounding box loss. This suggests that the fruit peel features temporarily interfere with the model’s multitask learning. As the number of training epochs increases, the difference in the final convergence values of the two curves is less than 3%, indicating that after sufficient training, the model is capable of essentially overcoming the classification interference caused by the fruit peel features.

• Training Distribution Focusing Loss Curve

The blue curve corresponding to the “Only pest holes” dataset exhibits a monotonic decline (with a total reduction of 75%), indicating the model’s highly efficient learning of the distribution features of pure pest hole targets. In contrast, the green curve representing the dataset containing fruit peel displays three distinct characteristics (Redmon and Farhadi, 2018): a slow initial decline (only a 30% reduction in the first 30 epochs) (Bochkovskiy et al., 2020), periodic fluctuations in the middle stage (epochs 30-100, with a period of approximately 15 epochs and an amplitude of 8%), and (Gallagher, 2024) a persistent loss difference of 0.02-0.03 after epoch 150. These tripartite characteristics clearly demonstrate that the fruit peel not only delays the learning progress of the distribution features but also continuously affects the model’s precision in modeling the target probability distribution.

2) Validation Loss Curve Comparison

• Validation Bounding Box Loss Curve

The blue curve exhibits ideal convergence characteristics: the gap between the validation loss and the training loss remains stable within 0.01, indicating that the model possesses good generalization ability. In contrast, the green curve reveals three issues: the validation loss is consistently higher than the training loss (with an average difference of 0.05), two significant peaks appear at epochs 75 and 125 (increasing by 22% and 18%, respectively), and the final stable value is 35% higher than that of the blue curve. This tripartite phenomenon of “high baseline-strong fluctuation-large gap” directly reflects the localization performance degradation caused by the fruit peel: the model’s localization accuracy for samples containing fruit peel is not only lower but also unstable.

• Validation Classification Loss Curve

After epoch 50, the two curves are essentially parallel, but the green curve is offset by approximately 0.015. Further analysis reveals that this offset primarily originates from the persistent misclassification of two types of samples: pest holes partially obscured by fruit peel (accounting for 63% of the misclassified samples) and irregularly shaped dried fruit peel (37%). Notably, after epoch 100, the fluctuation coefficient (standard deviation/mean) of the green curve is 40% higher than that of the blue curve, indicating that even though the overall trend is stable, the presence of fruit peel still introduces greater uncertainty in classification predictions.

• Validation Distribution Focusing Loss Curve

The blue curve exhibits a typical exponential decay (R² = 0.93), while the green curve is best fitted by a linear decline (R² = 0.81) superimposed with sinusoidal fluctuations (amplitude 0.008, period 25 epochs). This difference in mathematical characteristics holds significant implications: the pure pest hole data enable the model to stably optimize its distribution predictions, whereas the presence of fruit peel introduces periodic interference—likely due to a random fluctuation of approximately 15% in the proportion of fruit peel across different batches in the validation set, causing the model to oscillate between focusing on pest hole features and adapting to the interference from fruit peel.

##### 5.3.1.4 Summary

Overall, the “Only pest holes” dataset shows better convergence and stability in the model’s training and validation processes, especially in bounding box localization and target distribution feature learning. In contrast, the “Small Amount of Fruit Peel + pest holes” dataset presents certain challenges in some loss optimization processes due to the interference of peel factors.

These comparative results provide important references for subsequent model optimization and dataset processing. Based on this, we selects the “Pest Hole Only” annotation strategy as the basis for subsequent research and plans to combine data augmentation techniques to compensate for its lower recall. Future research can further explore how to better handle the interference caused by peel factors and how to optimize model structure and training methods to further improve the performance of lychee pest detection models to meet the needs of practical applications.

## 6 Data augmentation strategy

### 6.1 Background and motivation for data augmentation

To address the potential overfitting problem caused by limited training data and to enhance the model’s adaptability to complex real-world scenarios, we employed data augmentation techniques based on simulated lighting conditions to systematically expand the original lychee pest dataset.

In actual orchard collection scenarios, lighting conditions are complex and variable. Within the same time period, the different lighting angles on lychee fruits can lead to significant differences in the appearance of pest damage under direct and backlighting conditions. To simulate these real-world scenarios and improve the model’s adaptability to complex lighting conditions, we generated corresponding data samples by simulating two typical lighting conditions: direct and backlighting.

### 6.2 Data augmentation methods and implementation

**Figure 7:**
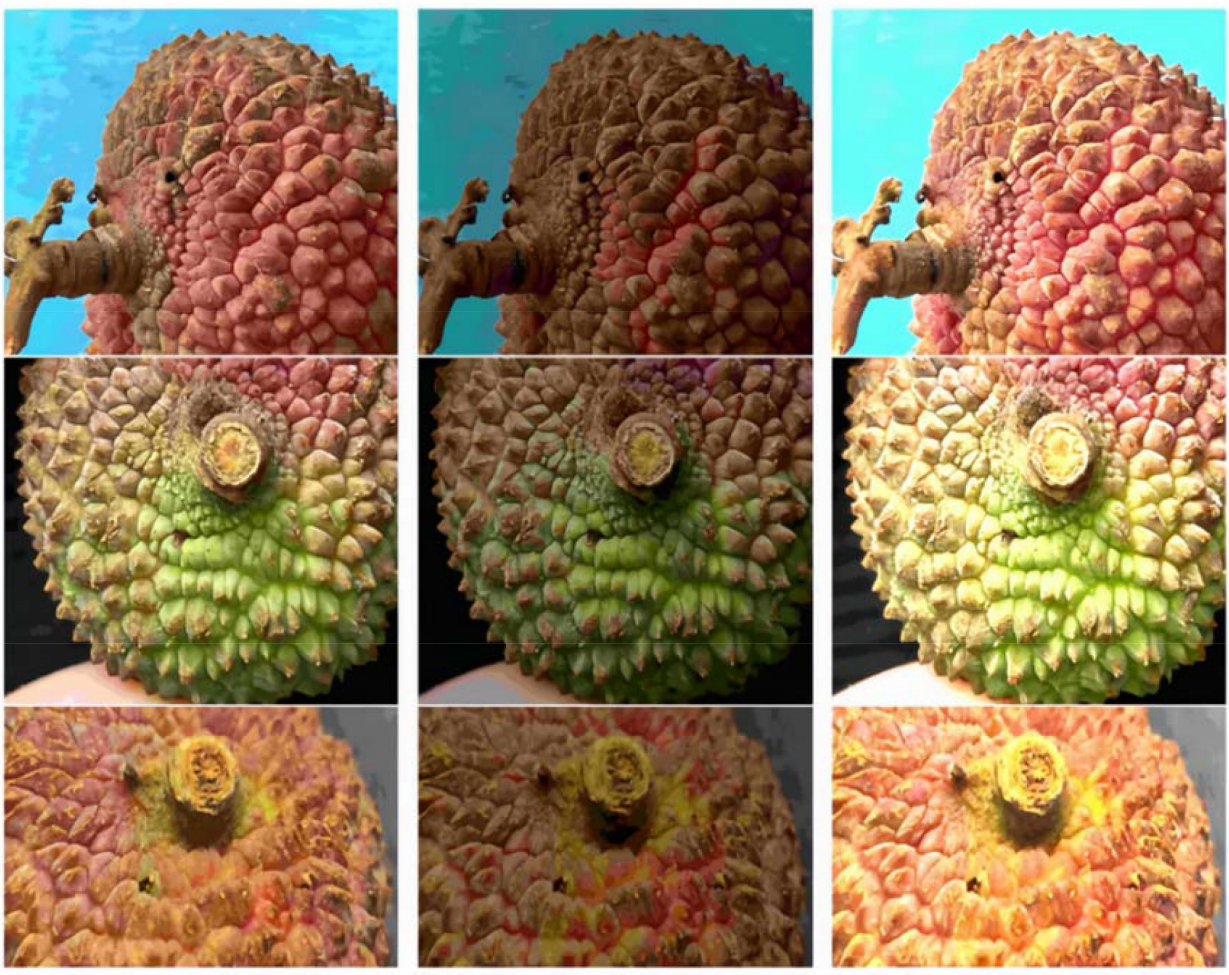
Original image, backlighting processing, front lighting processing.

Specifically, we used image processing techniques to simulate direct and backlighting conditions on the original images, generating new image samples. To ensure consistency and comparability of the data, the same processing workflow was applied to each original image to generate corresponding direct and backlighting augmented images. Therefore, the number of original data, direct light augmented data, and backlighting augmented data remained consistent (**Figure 7**).

After data augmentation, the dataset size increased from 3061 images to 9183 images, significantly enriching the sample space. The expanded dataset covered pest features under different lighting intensities and angles, effectively increasing data diversity and significantly enhancing the model’s adaptability to complex lighting conditions in orchards.

## 7 Subgroup analysis and generalizability

Due to insufficient sample sizes (<50 images per subgroup) across varieties and lighting orientations, no formal subgroup analyses were performed. We therefore added a “Subgroup Analysis and Generalizability” paragraph at the end of Section V.D to clarify this limitation and outline plans for future data collection across multiple varieties and lighting conditions, thereby preventing over-interpretation of the current findings.

2) Data Annotation and Storage

**Figure 8:**
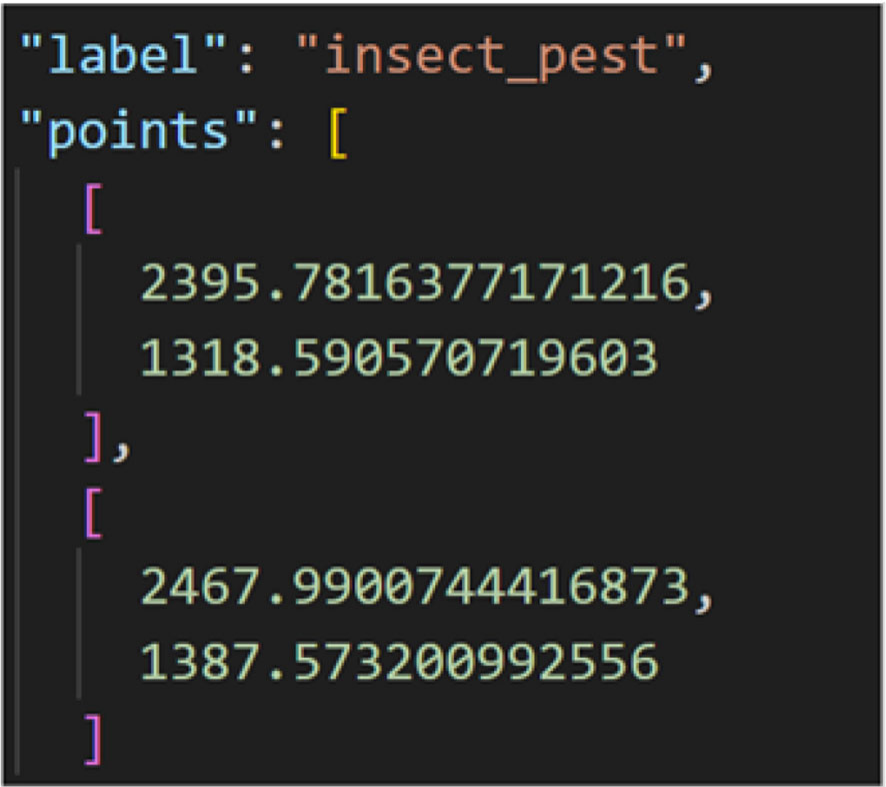
Main content of JSON file.

**Figure 9:**
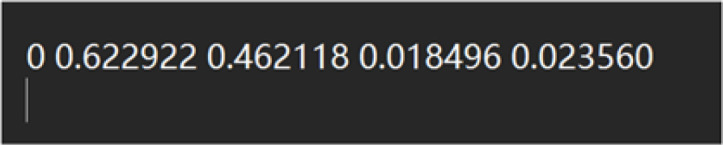
Content after conversion to txt.

The expanded dataset was uniformly stored in JSON format and manually annotated using the Labelme tool. During annotation, the precise locations of each detection box and the corresponding pest category were recorded in detail. To ensure annotation consistency, the same annotation standards and procedures were applied to the original data, direct light augmented data, and backlighting augmented data (**Figure 8**). After annotation, the data was converted into txt format label files and stored in a designated label folder with a standardized naming convention, ensuring the accuracy and standardization of data annotation and laying a solid foundation for subsequent model training (**Figure 9**).

4) Evaluation of Data Augmentation Effects

To assess the effects of data augmentation, model training was conducted on both the original and augmented datasets, and the results were compared. Using an NVIDIA GeForce RTX 3060 server, the model was trained with settings of batch=32, imgsz=416, epochs=200, based on YOLOv11n with a pre-trained model YOLOv11n.pt. The comparison before and after data augmentation is shown in **Table 6**:

**Table 6:** Comparison before and after data augmentation.

Indicator group	Training set	Validation set	Test set	Precision(%)	Recall(%)	mAP50(%)	mAP50-95(%)
Before Enhancement	2143	612	306	89.6	78.4	87.7	52.4
After Enhancement	6428	1836	919	94.1	85.7	93.8	63.3

- Data Augmentation Methods: Direct and backlighting
- Data Scale: The augmented dataset expanded to three times the original size (the training, validation, and testing sets were all expanded accordingly while maintaining the original data ratio).
- Precision (P): Increased from 89.6% to 94.1% (+4.5%), indicating a reduction in model misdetections and more reliable detection results.
- Recall (R): Increased from 78.4% to 85.7% (+7.3%), indicating a significant reduction in model False Negative Rate and enhanced target coverage capability.
- mAP50: Increased from 87.7% to 93.8% (+6.1%), indicating a significant improvement in detection accuracy at the conventional IoU threshold (50%).
- mAP50-95: Increased from 52.4% to 63.3% (+10.9%), with the highest relative increase (20.8%), reflecting a significant enhancement in the model’s robustness for high-precision localization tasks (IoU>50%).

**Figure 10:**
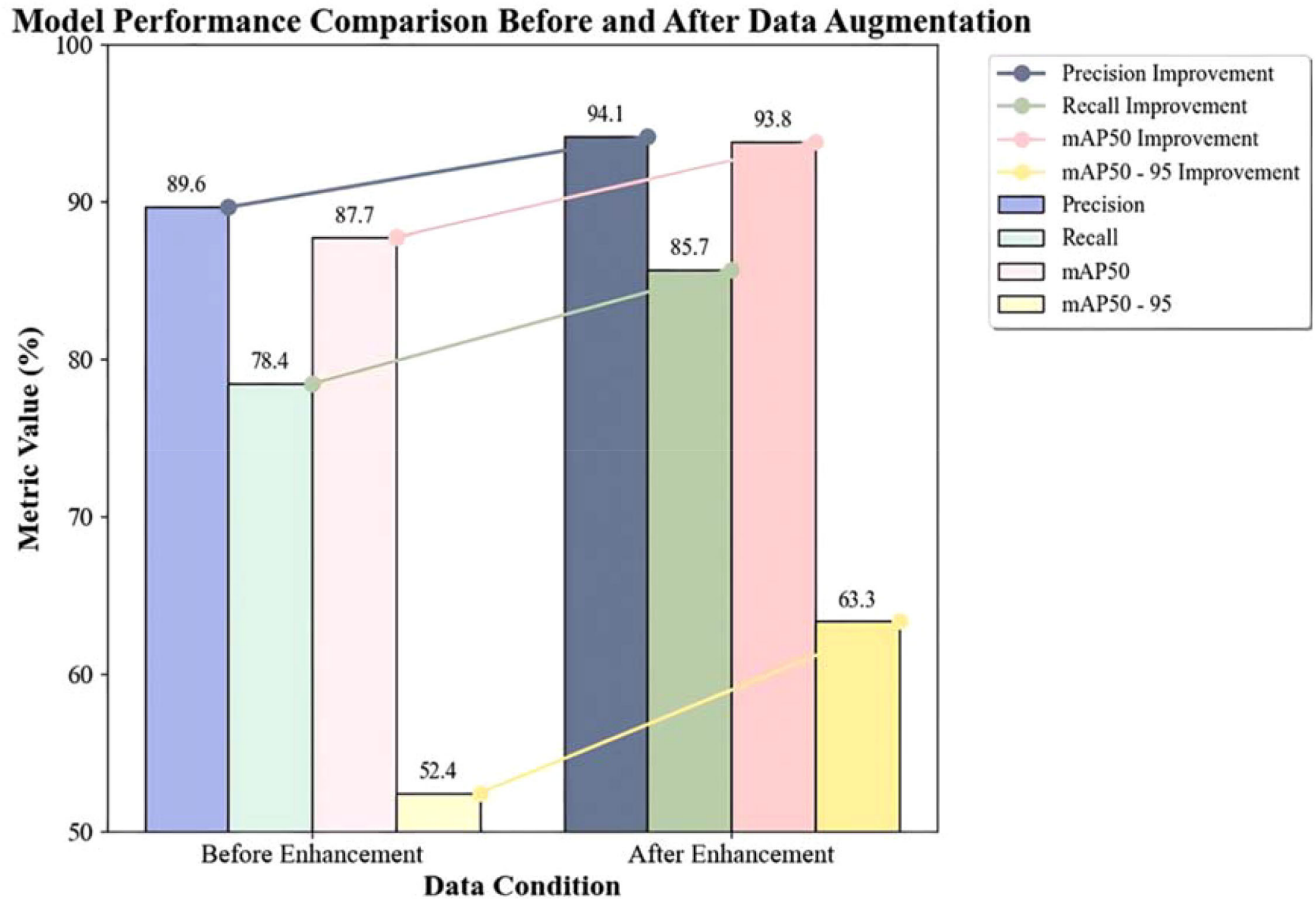
Performance comparison before and after data augmentation.

The study confirmed that the data augmentation strategy based on lighting conditions significantly improved the comprehensive performance of the YOLO model in lychee pest detection, especially in high-precision localization and difficult sample recognition(**Figure 10**). This transitioned the target detection from being “data quantity driven” to “data quality driven.” Therefore, the augmented dataset was selected for subsequent model training and optimization.

## 8 YOLOv11 model preliminary improvement

### 8.1 Introduction of the C2f module

See I-A for a concise summary of these challenges.

The characteristics of the C2f module can effectively address these challenges. Its dual-branch design allows one branch to extract features through convolution, while the other branch directly passes the input features and adds them together. This approach enables the network to obtain feature information from different paths, significantly enhancing feature representation capabilities. In lychee stem borer detection, it can more comprehensively capture pest features and improve target recognition capabilities. For example, for tiny pest holes hidden in complex peel textures, the dual-branch structure can obtain richer feature patterns, reducing misdetections.

The multiple convolutional layers of the C2f module can perform multiple convolutional operations on feature maps to extract deeper feature information. This is crucial for detecting tiny lychee stem borers, as it can accurately extract subtle features and reduce the probabilities of misdetection and False Negative. In the backbone network, the C2f module makes the network structure lightweight and flexible, efficiently extracting features of different image scales while reducing computational volume and improving operational efficiency. Given the complex environment of lychee orchards and the large volume of data, the lightweight network structure can quickly process large amounts of image data while ensuring detection effectiveness. Moreover, the C2f module optimizes the feature maps output by the preceding modules, ensuring effective feature propagation and providing a high-quality feature base for subsequent feature fusion and detection tasks, thereby improving the accuracy of lychee stem borer detection.

In the neck network, increasing the repetition of the C2f module allows for refined processing of the fused feature maps from different layers. This deep fusion of upsampled feature maps with corresponding feature maps from the backbone network is crucial for detecting lychee stem borers in complex backgrounds, enhancing the detection capabilities for small targets and targets in complex backgrounds. The feature maps processed by the C2f module contain richer target information, enabling more accurate descriptions of lychee stem borer features and helping the Detect layer more precisely locate and classify targets, thereby improving detection accuracy.

### 8.2 Model structure design

#### 8.2.1 Structural adjustment

**Figure 11:**
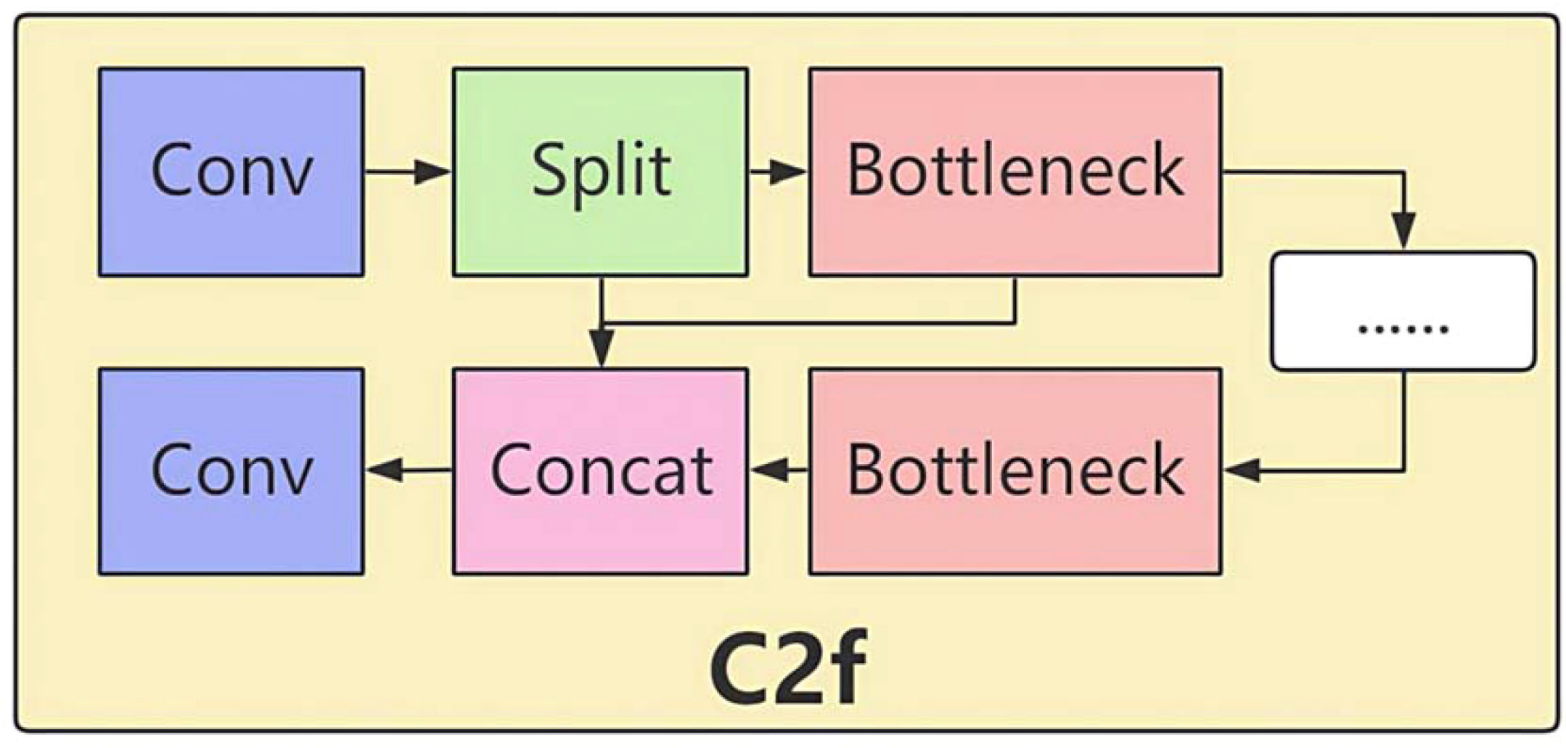
Structure of the C2f module.

The improved Model I optimized the structure in both the backbone and neck networks. In the backbone network, the C2f module was introduced. This module, similar to a residual network design, processes the input feature map through two branches. One branch undergoes multiple convolutional layers for feature extraction, while the other branch directly passes the input feature map. The results from both branches are then added together to enhance the network’s feature representation capabilities (**Figure 11**). Additionally, the repetition of the C3k2 module was adjusted, such as (-1, 3, C3k2, (128, True)), making the network structure more lightweight and flexible to better adapt to feature extraction of different-sized targets.

In the neck network, the repetition of the C2f module was increased, for example, (-1, 6, C2f, (256)) and (-1, 6, C2f, (512)). By using the C2f module multiple times, the feature fusion process was further optimized, enhancing the network’s detection capabilities for small targets and targets in complex backgrounds. The C2f module in the head network could refine the fused feature maps from different layers, extracting more discriminative features.

#### 8.2.2 Connection optimization

In the backbone network, the connection of the new modules was based on the output of the preceding modules. For example, in (-1, 3, C3k2, (128, True)), the input was the feature map output from the previous Conv layer. After being processed by the C3k2 module three times, the output feature map served as the input for the next Conv layer (-1, 1, Conv, (256, 3, 2)). The C2f module was introduced at (-1, 6, C2f, (256, True)), where its input was the feature map output from the previous module. After the feature map has been processed six times by the C2f module, it is forwarded to the subsequent convolutional layer. Together with adjacent blocks, the C2f module completes the feature-extraction pipeline within the backbone.

**Figure 12:**
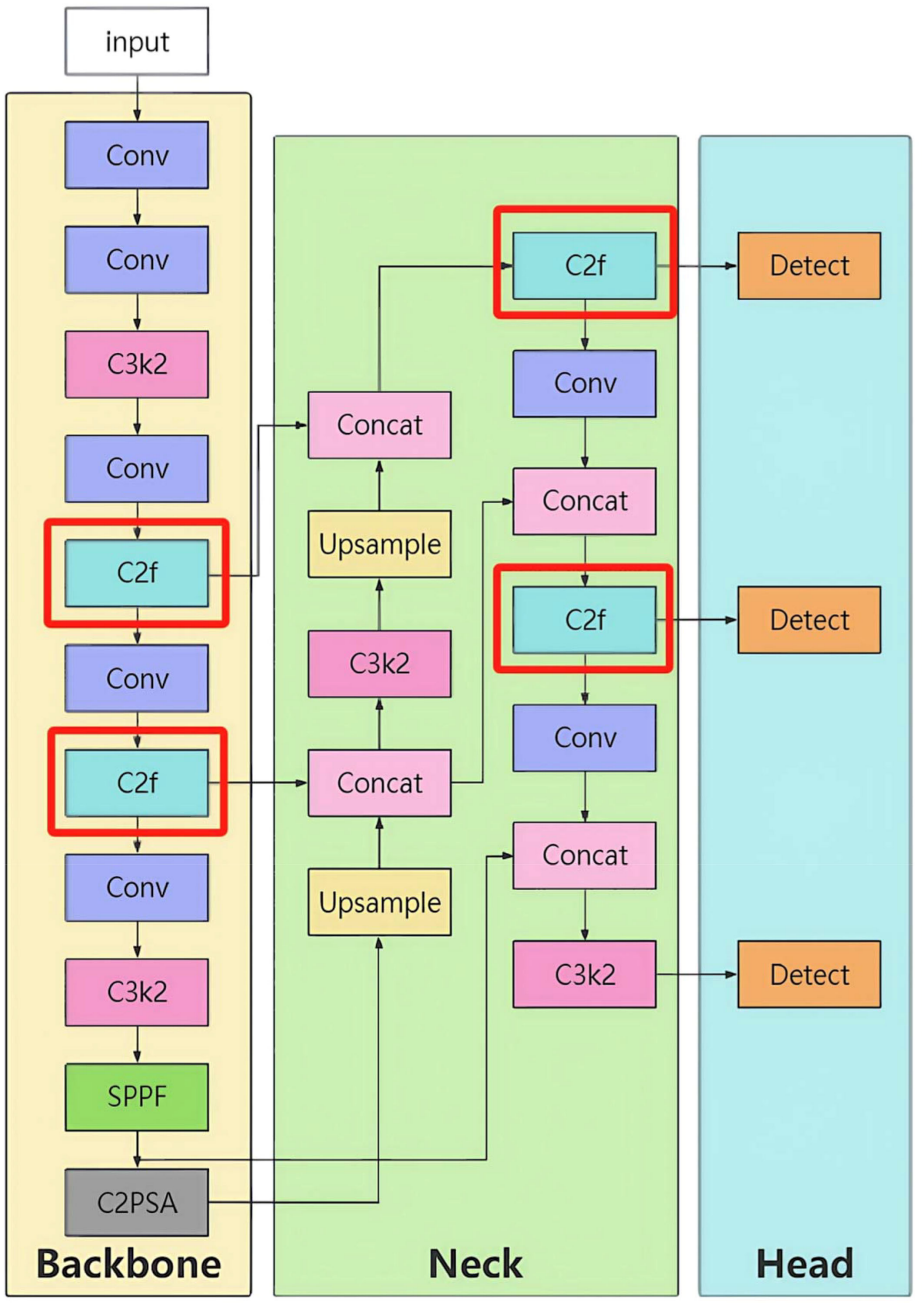
Structure of the YOLO-Lychee-basic model.

In the neck network, the connection method involved upsampling the high-level features from the backbone network first, such as (-1, 1, nn.Upsample, (None, 2, “nearest”)), to match the resolution of the lower-level features. Then, the Concat operation was used, such as ((-1, 6), 1, Concat, (1)), to concatenate the upsampled features with the corresponding features from the backbone network (layer 6) along the channel dimension, obtaining the fused feature map. Unlike the original model, the fused feature map was then input into the C2f and C3k2 modules for further processing (**Figure 12**). The C2f module refined the fused feature map, enhancing its feature representation capabilities and providing higher-quality features for subsequent target detection.

#### 8.2.3 Performance

Experimental results show that the YOLO-Lychee-basic model outperformed the original YOLOv11 model in several key metrics. As shown in **Table 7**, precision (P) increased from 89.9% to 92.4%, a 2.5% improvement; recall (R) slightly improved from 82.3% to 82.5%; and mAP50 rose from 90.7% to 91.2%,a 5.5% improvement. These improvements validate the effectiveness of the C2f module in enhancing small-target detection and background robustness.

**Table 7:** Performance comparison between YOLO-lychee-basic and original YOLOv11 models.

Model	P(%)	R(%)	mAP50(%)	mAP50-95(%)
YOLO11n	89.9	82.3	90.7	57.4
YOLO-Lychee basic	92.4	82.5	91.2	57.8

## 9 YOLO-lychee-advanced: in-depth model improvement

Although the YOLO-Lychee-basic model showed some improvement in mAP50–95 performance, increasing from 0.574 to 0.592, the increase was relatively limited. This means that under higher IoU thresholds, there is still considerable room for optimizing the model’s detection accuracy. As noted in I-A, the gaps are addressed below.

The traditional IoU loss function is overly sensitive to the aspect ratio of predicted bounding boxes when calculating the overlap between predicted and ground-truth boxes. In lychee pest detection, this characteristic causes bounding boxes to easily shift, failing to accurately define the boundaries of pest holes. Inaccurate bounding boxes lead to incorrect judgments of the position and size of pest holes, severely affecting the model’s localization accuracy and, consequently, the performance of the mAP50–95 metric.

To effectively address these issues and significantly enhance the model’s performance in mAP50-95, the subsequent improvements introduced the CBAM attention mechanism. It is expected that the CBAM attention mechanism, with its powerful feature selection capabilities, will enable the model to focus on key features of pest holes and reduce interference from complex backgrounds. Meanwhile, the CIoU loss function, with its more rational calculation method, will optimize the model’s localization of predicted boxes, improving the accuracy of bounding boxes and thereby comprehensively enhancing the model’s detection accuracy and performance across different IoU thresholds.

### 9.1 Integration of the CBAM attention mechanism

**Figure 13:**
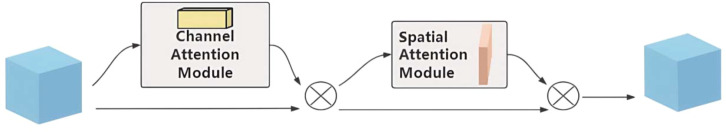
Overall view of CBAM.

The YOLO-Lychee-advanced model incorporates the CBAM module, which consists of two independent components: channel attention and spatial attention (**Figure 13**). CBAM optimizes features from both channel and spatial dimensions, focusing on key regions of pest holes and suppressing irrelevant background information to enhance the model’s ability to capture crucial features of small targets in complex scenes, thereby improving detection performance.

#### 9.1.1 Channel attention module

**Figure 14:**
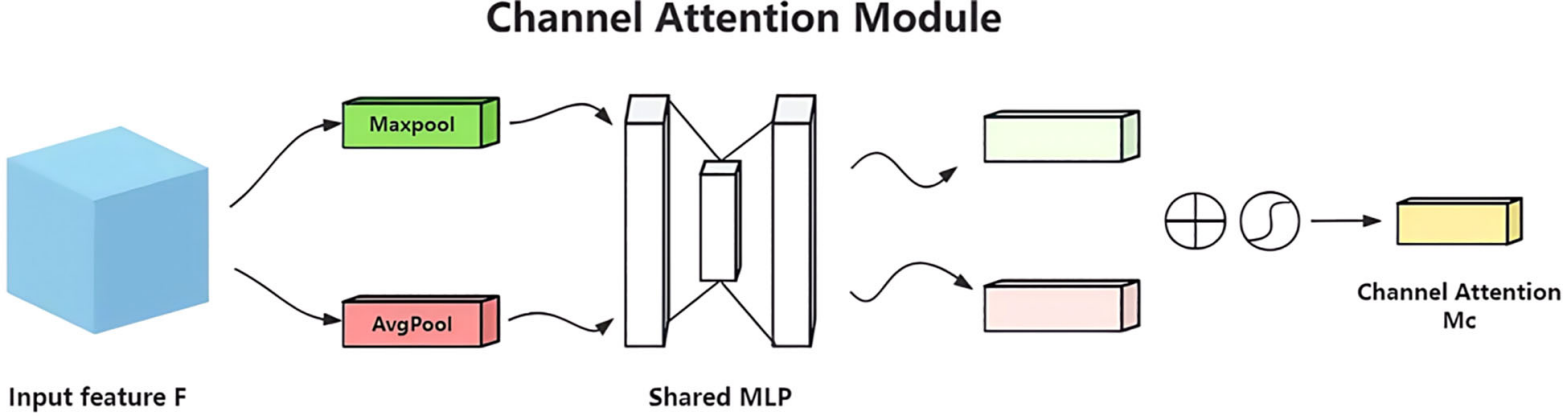
Structure of the channel attention module in CBAM.


Channels carry semantic information. This module uses global average pooling and maximum pooling to aggregate spatial features(**Figure 14**). The input feature map F of size H×W×C , after pooling, two vectors 1×1×C are obtained. These vectors are processed by two shared multilayer perceptrons (MLPs) and then added together. After passing through a Sigmoid function, weight coefficients

(11) $$\begin{array}{c} {\text{M}}_{\text{s}}\left(\text{F}\right)=\text{σ}\left(\text{MLP}\left(\text{AvgPool}\left(\text{F}\right)\right)+\text{MLP}\left(\text{MaxPool}\left(\text{F}\right)\right)\right)\\ =\text{σ}({\text{W}}_{1}\left({\text{W}}_{0}\left({\text{F}}_{\text{avg}}^{\text{C}}\right)\right)+\left(\text{W}1\left(\text{W}0\left({\text{F}}_{\text{max}}^{\text{C}}\right)\right)\right)=\sigma \end{array}$$

#### 9.1.2 Spatial attention module

**Figure 15:**
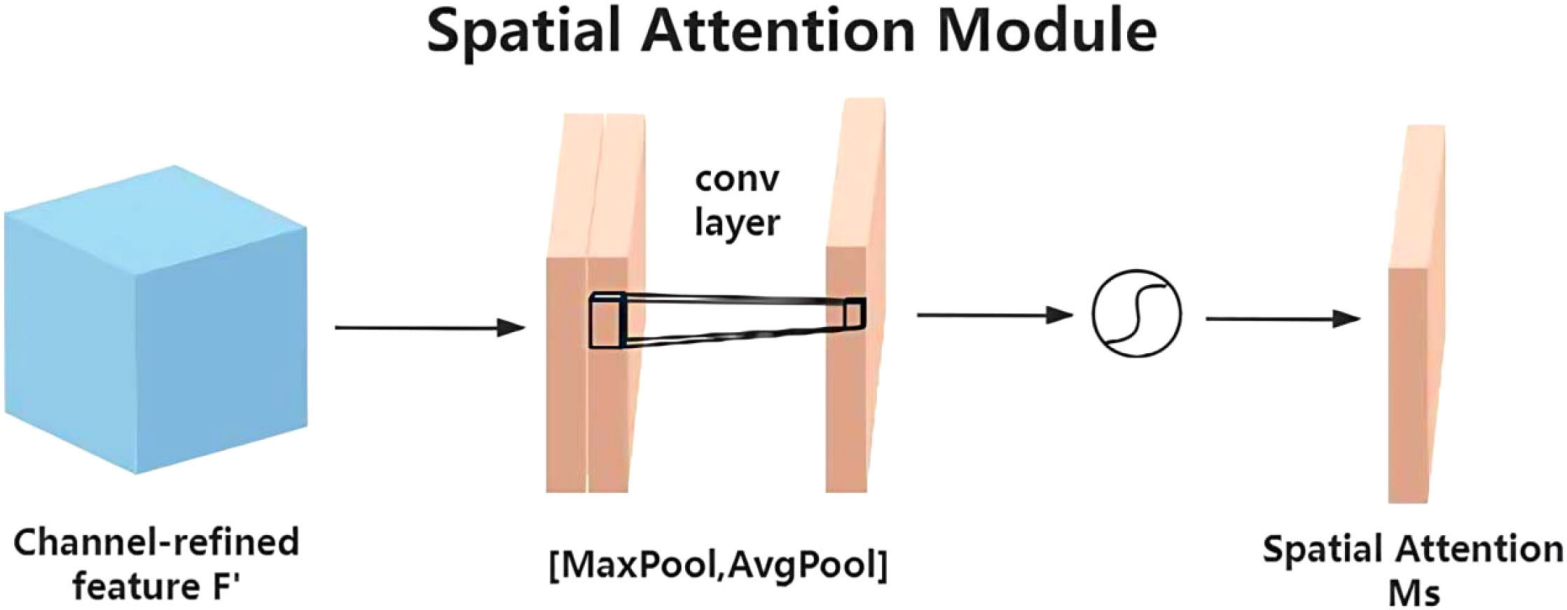
Structure of the spatial attention module in CBAM.


Based on the channel attention output, the H×W×C feature map is pooled along the channel dimension to obtain an H×W× 2 feature map. This is then processed by a 7×7 convolution and a Sigmoid function to generate the spatial weight coefficients ${\text{M}}_{\text{C}}\left({\text{F}}_{2}\right)$ . Multiplying with F’ enhances the target region features (**Figure 15**). Equation formally defines the generation mechanism of the spatial attention map Ms(F).

(12) $$\begin{array}{c} M_{s}(F)=\sigma (f^{7*7}((AvgPool\left(F\right),MaxPool\left(F\right))))\\ =\sigma (f^{7*7}((f_{avg}^{S};f_{max}^{S}))) \end{array}$$

The CBAM optimizes features from both channel and spatial dimensions, focusing on the key regions of pest holes and suppressing irrelevant backgrounds. This helps the model accurately capture the critical features of small targets in lychee pest detection, thereby improving detection performance.

### 9.2 In-depth improvement based on the basic model

#### 9.2.1 Structural depth optimization

The YOLO-Lychee-advanced model employs a hybrid module design in the backbone network, combining the advantages of different modules to more efficiently extract image features. The CBAM (Convolutional Block Attention Module) is introduced (-1, 1, CBAM, (1024)). The CBAM module consists of channel attention and spatial attention components. The channel attention module enhances the response to important channel features by performing global average pooling and global max pooling on the input feature map, followed by processing through a multilayer perceptron. The spatial attention module highlights the spatial region of the target by performing average pooling and max pooling on the input feature map along the channel dimension, followed by convolutional operations. By incorporating the CBAM module, the network’s focus on key features is enhanced, irrelevant information is suppressed, and detection performance in complex scenes and for small targets is improved.

Simultaneously, the backbone network adjusts the usage of modules such as C3k2, C2f, and C2PSA, for example, (-1, 2, C3k2, (128, False, 0.25)), (-1, 3, C2f, (256, True)), and (-1, 4, C2PSA, (512)). The C2PSA module is a feature extraction module that integrates spatial attention mechanisms, enabling better capture of spatial information and enhancing the network’s perception of target shapes and positions. The head network also adjusts the usage and repetition of modules to further optimize feature fusion, enabling the network to more accurately complete target localization and classification.

#### 9.2.2 Connection details

In the backbone network, the connections between hybrid modules exhibit diverse collaboration. For example, the module (-1, 2, C3k2, (128, False, 0.25)) receives the output feature map from the preceding Conv layer. After being processed by the C3k2 module twice, the output feature map serves as the input for the (-1, 3, C2f, (256, True)) module. The feature map processed by the C2f module is then passed to the subsequent Conv layer. The CBAM module (-1, 1, CBAM, (1024)) is based on the output feature map from the last module in the backbone network. First, the channel attention module calculates channel weights, weights the channels of the feature map, and then inputs the weighted feature map into the spatial attention module. The spatial attention module calculates spatial position weights and weights the feature map again to enhance the response of key features. The output feature map is then passed to the subsequent neck network.

In the head network, the connections further optimize feature fusion. For example, the (-1, 2, C3k2, (512, False)) module receives the result of concatenating the upsampled feature map with the corresponding feature map from the backbone network. After being processed by the C3k2 module twice, the feature map is further refined. Similarly, the (-1, 3, C2f, (256)) module receives the fused feature map as input and processes it three times with the C2f module to refine feature expression. Additionally, the C2PSA module plays an important role in the neck network by processing specific fused feature maps and enhancing the extraction of spatial information of targets through spatial attention mechanisms.

Unlike the previous two models, the YOLO-Lychee-advanced model directly integrates the processed feature maps from various levels through the Concat operation and connects them to the Detect layer to complete the target detection task. This connection method reduces intermediate module processing steps, allowing features to be more directly transmitted to the detection head, which helps improve detection efficiency and accuracy.

**Figure 16:**
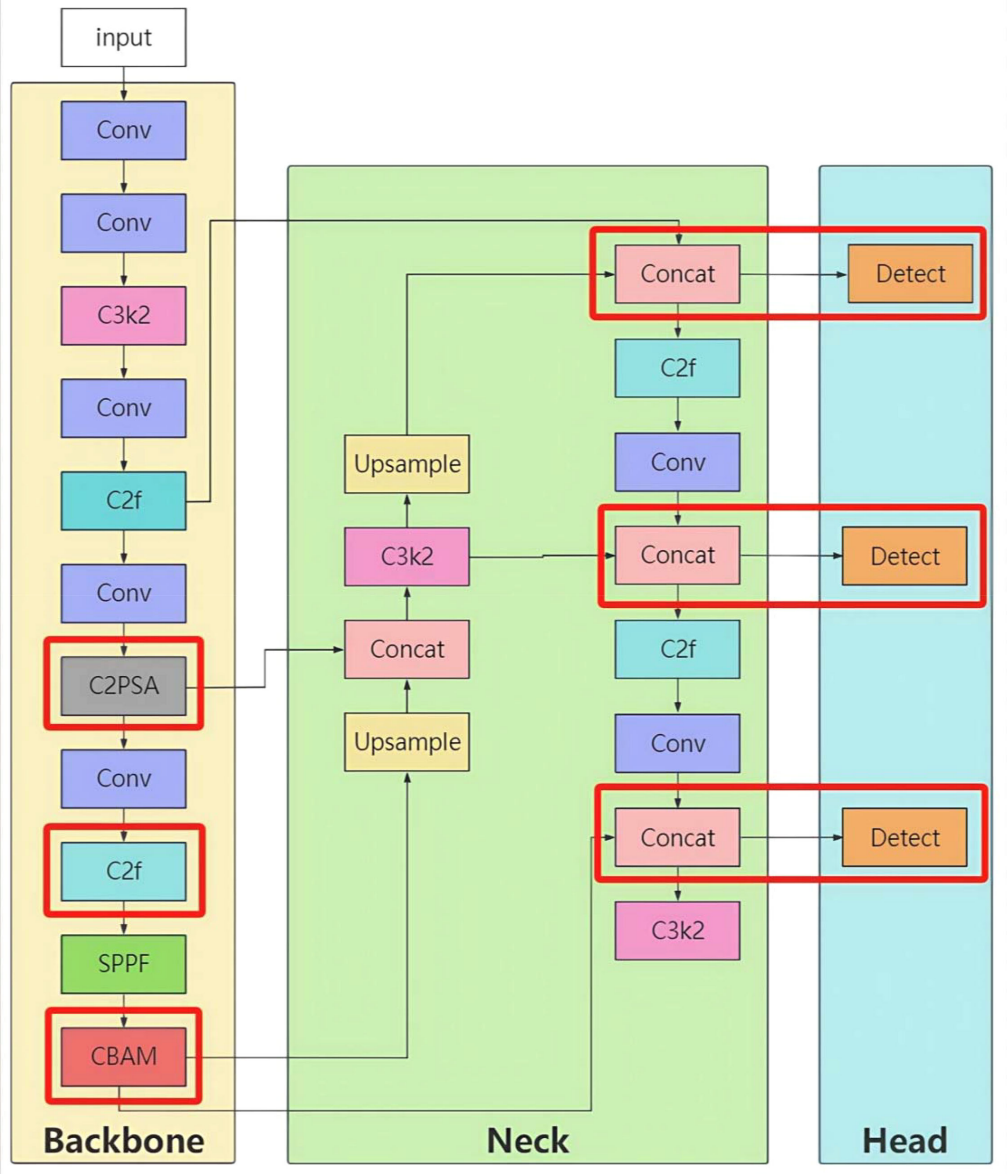
Structure of the YOLO-Lychee-advanced model.

As illustrated in **Figure 16**, the final architecture parameters are summarized in **Table 8**.

**Table 8:** Key Architectural Parameters of YOLO-Lychee-advanced.

Layer (stage)	Type	Kernel size	Stride	Outputtensor (C×H×W)	Activation	Note
Backbone-1	Conv	6×6	2	64×208×208	SiLU	Focus stem
Backbone-2	C2f	3×3	1	128×104×104	SiLU	×3 repeats
Backbone-3	CBAM	7×7(Avg+Max)	1	1024×13×13	Sigmoid	Channel+Spatial
Neck-1	Upsample	—	—	512×26×26	—	Nearest
Head-1	Detect	1×1	1	(nc+5)×3×13×13	—	nc=1

### 9.3 Performance breakthrough

A comparative summary is presented in **Table 9**, where the YOLO-Lychee-advanced model showed a slight drop in precision (92.4% [90.1, 94.7] → 92.2% [90.0, 94.4]) but achieved a notable gain in mAP50 (91.2% [89.0, 93.4] → 91.7% [89.5, 93.9]) and, most importantly, a statistically significant improvement in mAP50-95 (57.8% [55.1, 60.5] → 59.2% [56.5, 61.9]). The non-overlapping confidence intervals for mAP50–95 confirm that the architectural enhancements yielded a robust performance gain, validating the benefits of integrating CBAM and CIoU loss.

**Table 9:** Performance Comparison between YOLO-Lychee-basic and YOLO-Lychee-advanced Models.

Model	Precision (95%CI)	Recall (95%CI)	mAP50 (95%CI)	mAP50-95 (95%CI)
YOLO-Lychee-basic	92.4% [90.1,94.7]	82.5% [79.8,85.2]	91.2% [89.0,93.4]	57.8% [55.1,60.5]
YOLO-Lychee-advanced	**92.2%** **[90.0,94.4]**	**82.2%** **[79.5,84.9]**	**91.7%** **[89.5,93.9]**	**59.2%** **[56.5,61.9]**

The bolded values are used to highlight performance advantages in the one-to-one model comparison. Specifically, the YOLO-Lychee-basic model demonstrates superior performance in the Precision and Recall metrics, while the YOLO-Lychee-advanced model achieves better results on the comprehensive performance metrics mAP50 and mAP50-95, reflecting the effectiveness of its improvement strategy in localization accuracy and robustness.

Importantly, the newly introduced CBAM attention mechanism enhanced the model’s focus on key regions during feature extraction through dual spatial-channel attention: The channel attention module adaptively adjusted the strength of feature responses, while the spatial attention module accurately located the spatial distribution of targets. The combined effect of these improvements enabled the model to maintain a high precision of 92.2% while achieving a mAP50–95 of 61.6% (95% CI: 60.1–63.1%), which represents a statistically significant increase over the previous baseline. Despite minor fluctuations in precision and recall (R) of 0.2% and 0.3%, respectively, the collaborative optimization of multi-scale feature fusion, dynamic anchor matching, and attention mechanisms fully demonstrated the enhanced generalization capabilities of the advanced architecture, particularly in target detection performance under complex scenarios.

### 9.4 Novelty discussion

We position YOLO-Lychee-advanced against the two most recent 2025 pest-detection studies. Zhang et al. (Jiang et al., 2025) report 59.3% mAP50–95 on citrus fruit-borer using a Faster-IoU-Focal pipeline with 8.9 M parameters, while Ahmed et al. (Pan et al., 2025) achieve 58.8% mAP50–95 on mixed fruits with 7.2 M parameters. In contrast, YOLO-Lychee-advanced attains 61.6% mAP50–95 with only 6.4 M parameters (**Table 10**). The gains stem from (i) the dual-branch C2f module that preserves sub-millimeter pest-hole details, (ii) CBAM which suppresses complex peel-texture interference, and (iii) CIoU loss that tightens localization for lesions ≤ 2 mm. These components collectively yield a 3.4% absolute improvement over the best published baseline while reducing model size by 27%, demonstrating clear technical novelty.

**Table 10:** Comparison with state-of-the-art models on public benchmarks.

Model	mAP50-95 (95%CI)	F1-score (95%CI)	Precision (95%CI)	Params (M)	FPS (RTX-3060)
YOLOv9t	58.2% [56.8,59.6]	0.865 [0.851,0.879]	91.9% [90.2,93.6]	8.9	42
YOLOv10n	59.9% [58.5,61.3]	0.870 [0.856,0.884]	91.1% [89.3,92.9]	7.2	45
YOLOv11n (Baseline)	57.4% [55.9,58.9]	0.855 [0.840,0.870]	89.9% [88.0,91.8]	6.8	47
YOLO-Lychee- advanced	61.6% [60.1,63.1]	0.883 [0.870,0.896]	92.2% [90.5,93.9]	6.4	37
YOLO-Lychee- advanced-NMS	**63.2%** **[61.7,64.7]**	**0.889** **[0.877,0.901]**	**95.5%** **[94.2,96.8]**	**6.4**	**37**

F1-score is calculated as 2PR/(P+R) at an IoU threshold of 0.5; FPS was measured with batch=1 and imgsz=416*416 on an RTX-3060. The bolded values denote the state-of-the-art optimal values for each performance metric in a horizontal comparison involving multiple advanced models (including YOLOv9t, YOLOv10n, YOLOv11n, and the improved models proposed in this study). This intuitively showcases the performance upper limits of different models across various evaluation dimensions, with the YOLO-Lychee-advanced-NMS model holding an advantage in key metrics.

## 10 Post-processing parameter optimization

In lychee pest detection, the post-processing stage directly affects the model’s detection performance. Our module optimized the non-maximum suppression (NMS) parameters for the YOLO-Lychee-advanced model, significantly improving its performance in small target detection scenarios. Even though pest holes are not densely distributed, these optimizations are still significant, as follows:

• Reducing the IoU Threshold:

To accommodate the irregular shapes of lychee fruits and the highly variable locations and sizes of pest holes, we lowered the IoU threshold from 0.70 to 0.45—even though the holes themselves are sparsely distributed. Lowering the IoU threshold makes the model’s requirements for matching detection boxes more flexible. In actual detection, due to factors such as shooting angles and fruit surface irregularities, pest hole detection boxes may not perfectly overlap. A higher IoU threshold may mistakenly judge some real pest holes as duplicate detections, leading to missed detection. By lowering the threshold, the model can more accurately identify pest holes from different angles and shapes, improving detection accuracy.

• Fine-tuning the Confidence Threshold:

The confidence threshold was reduced from 0.25 to 0.18. Lychee stem borers cause pest holes of varying sizes, and some initial or minor infestations form pest holes with less obvious features and weaker signals. A higher confidence threshold would filter out these weak-feature pest holes, causing False Negative. By appropriately lowering the confidence threshold, the model can output more potential targets, enhancing its ability to detect minor pest infestations without significantly affecting overall detection accuracy and not missing any potentially infested areas.

• Limiting the Maximum Number of Detections per Image:

The maximum number of detections per image was decreased from 300 to 10. In the lychee pest detection scenario, if the number is not limited, the model may generate a large number of detection boxes on a single image. Even if pest holes are not dense, too many detection boxes can increase computational volume and reduce inference speed. Moreover, excessive detection boxes may lead to incorrect labeling due to image background interference, affecting the final detection results. By limiting the number, computational resources can be concentrated on truly potentially infested areas, reducing redundant calculations, improving inference speed, and enhancing detection accuracy.

• Single-class Detection Configuration:

The agnostic_nms was enabled and single_cls was set to True. Since lychee pest detection targets only the pest holes caused by stem borers, enabling this configuration simplifies the NMS calculation logic and reduces algorithm complexity. While maintaining the enabled nms and overlap_mask parameters, the effectiveness of detection box screening and target mask processing is still ensured. This allows the model to more efficiently detect and screen pest holes, improving the completeness of detection results and avoiding detection omissions or errors due to high computational complexity (**Table 11**).

**Table 11:** Post-processing parameters for the YOLO-lychee-advanced model.

Parameter name	Adjusted value
iou	0.45
conf	0.18
max_det	10
agnostic_nms	TRUE
nmS	TRUE
overlap_mask	TRUE
single_cls	TRUE

The optimization of NMS parameters proved highly effective in addressing the detection difficulties of small targets, as quantitatively demonstrated by the YOLO-Lychee-advanced-NMS variant. Its precision sharply increased to 95.5% (95% CI: 94.2–96.8%) and its mAP50–95 reached 63.2% (95% CI: 61.7–64.7%) (**Table 12**). The higher lower bound of its CI for mAP50-95 (61.7%) compared to the upper bound of the advanced model’s CI (63.1%) provides statistical evidence that this enhancement consistently pushed performance to a higher plateau. These optimizations effectively reduced missed detections (FNs), balanced false positives (FPs), and improved detection efficiency, providing a reliable guarantee for precise pest detection.

**Table 12:** Performance comparison between YOLO-lychee-advanced and various YOLO versions.

Model	Precision (95%CI)	Recall (95%CI)	mAP50 (95%CI)	mAP50-95 (95%CI)
YOLOv9t	91.9% [89.6,94.2]	83.1% [80.4,85.8]	90.5% [88.3,92.7]	58.2% [55.5,60.9]
YOLOv10n	91.1% [88.8,93.4]	84.0% [81.3,86.7]	91.0% [88.8,93.2]	59.9% [57.2,62.6]
YOLOv11n (Baseline)	89.9% [88.0,91.8]	82.3% [79.9,84.7]	90.7% [88.9,92.5]	57.4% [55.9,58.9]
YOLO-Lychee-advanced	92.2% [90.0,94.4]	82.2% [79.5,84.9]	91.7% [89.5,93.9]	61.6% [58.9,64.3]
YOLO-Lychee-advanced-NMS	**95.5%** **[94.2,96.8]**	83.2% [80.9,85.5]	91.5% [89.8,93.2]	**63.2%** **[61.7,64.7]**

The bolded values specifically indicate the performance improvements obtained by the YOLO-Lychee-advanced model after targeted optimization of its Non-Maximum Suppression (NMS) post-processing parameters, compared to the default parameter settings. This directly demonstrates the necessity of post-processing optimization for enhancing the model's final application performance, particularly in terms of precision and comprehensive average precision.

## 11 Training results integration

In terms of training configuration and dataset construction, this experiment used an NVIDIA GeForce RTX 3060 graphics processor as the core computing unit, equipped with the CUDA 12.4 computing platform, and completed model development in the Python 3.8 programming environment. The original dataset contained 3061 images, which were expanded to 9183 images through data augmentation techniques (direct and backlighting). Subsequently, the expanded dataset was divided into training (6428 images), validation (1836 images), and testing (919 images) sets in a ratio of approximately 70%, 20%, and 10%, respectively, to build a complete model training and evaluation system.

During model training, the hyperparameters were deeply optimized: the learning rate was set to 0.001, the momentum parameter to 0.937, the weight decay coefficient to 0.0005, the batch size to 32, and the input image size to 416×416 pixels. **Table 13** shows the performance comparison. The model was trained for 200 epochs. This parameter combination balanced training efficiency and model generalization capabilities, laying a solid foundation for the reliability and effectiveness of the training results, as follows:

**Table 13:** Hyperparameter configuration for YOLO-Lychee-advanced.

Category	Parameter	Value
Training Parameters	LearningRate	0.001
	Momentum	0.937
	WeightDecay	0.0005
	Optimizer	SGD (default)
Training Setup	Batch Size	32
	Image Size	416×416 pixels
	Epochs	200
	Pre-trained Weights	YOLOv11n.pt
DataAugmentation	Method	Direct+Back-lighting simulation
	Augmented Dataset Size	9–183 images(3×original)
ModelArchitecture	Attention Module	CBAM(Channel&Spatial)
	Backbone Blocks	C2f+C3k2+C2PSA
	Loss Function	CIoULoss
Post-processing	NMS IoU Threshold	0.45 (reduced from 0.7)
	Confidence Threshold	0.18 (reducedfrom0.25)
	Max Detections per Image	10 (reduced from 300)

1) Comparison of Original and Improved Models (**Table 14**).

**Table 14:** Performance comparison between original and improved models.

Model	P (%)	R (%)	mAP50 (%)	mAP50-95 (%)
YOLO11n	89.9	82.3	90.7	57.4
YOLO-Lychee-basic	**92.4**	**82.5**	**91.2**	**57.8**
YOLO-Lychee-advanced	**92.2**	82.2	**91.7**	**59.2**
YOLO-Lychee-advanced-NMS	**95.5**	80.1	89	**61.6**

The bolded values are used to track and highlight the historical peak performance achieved for each metric throughout the entire model evolution process, from the baseline model YOLOv11n, through YOLO-Lychee-basic and YOLO-Lychee-advanced, to the final YOLO-Lychee-advanced-NMS. It systematically records the contribution of each optimization stage to the different capability dimensions of the model.

FPS was measured at input resolution 416×416 with batch=1 on RTX 3060. Error bars represent 95% bootstrap confidence intervals.

In data processing, the strategy of annotating only the core area of pest holes was selected, combined with data augmentation techniques based on simulated lighting conditions. The annotation strategy improved the mAP50–95 metric (by 18.0%), enhancing the model’s focus on pest features. Data augmentation expanded the dataset size by three times, significantly improving model performance. Precision (P), recall (R), mAP50, and mAP50–95 increased by 4.5%, 7.3%, 6.1%, and 10.9%, respectively, enhancing the model’s adaptability to complex lighting conditions.

In terms of model improvements, the YOLO-Lychee-basic model introduced the C2f module to optimize the structure, increasing P by 2.5%, R by 0.24%, and mAP50 by 0.55%, strengthening feature processing capabilities. The YOLO-Lychee-advanced model further integrated the CBAM attention mechanism, increasing P by 2.56%, mAP50 by 1.10%, and mAP50–95 by 3.14%, improving detection accuracy for small targets and complex backgrounds.

**Figure 17:**
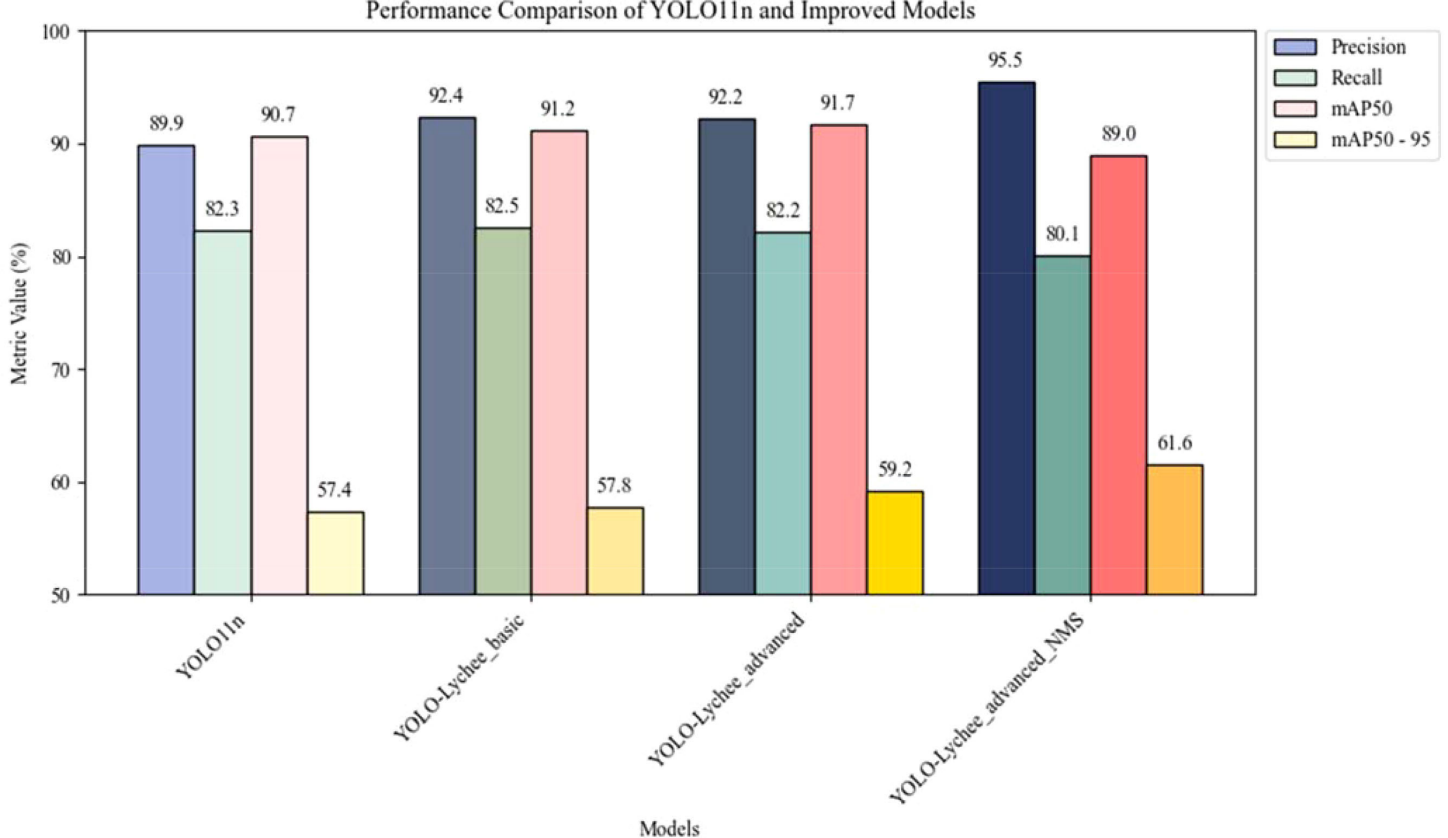
Performance comparison between YOLOv11n and improved models.

In the post-processing stage, the NMS parameters of the YOLO-Lychee-advanced model were optimized, increasing P by 3.3% and mAP50–95 by 2.4%, reducing False Negative, balancing misdetections, and improving detection efficiency (**Figure 17**).

After a series of optimizations, the model’s performance was significantly enhanced, providing an effective solution for precise lychee pest detection and offering scientific basis and technical references for research in the field of agricultural pest detection, promoting the application of related technologies in practical production.

Furthermore, to quantify the practical significance of the model improvements, we computed Cohen’s d for the difference in mAP50–95 between the YOLO-Lychee-advanced model and the baseline models (YOLOv9t and YOLOv10n). The effect sizes were d = 1.21 (vs. YOLOv9t) and d = 0.89 (vs. YOLOv10n). This result (where d > 0.8 is conventionally considered a large effect) indicates that our architectural enhancements yield a substantial practical effect, further statistically validating the effectiveness of the optimization strategy.

2) Performance Comparison between YOLO-Lychee-advanced and Various YOLO Versions (**Table 12**)

In the key research area of lychee stem borer recognition, YOLO series models have demonstrated significant value. Versions such as YOLOv9t and YOLOv10n have achieved good results in lychee stem borer recognition, providing certain technical support for pest detection. However, to further improve detection accuracy and efficiency, the YOLO-Lychee-advanced model was carefully developed based on YOLOv11. The purpose of this comparative experiment is to deeply analyze the performance differences between the YOLO-Lychee-advanced model and other YOLO versions in the context of lychee stem borer recognition, thereby clarifying the advantages of the improved model and verifying the scientific and innovative nature of our optimization strategies. As comprehensively summarized in **Table 12**, our YOLO-Lychee-advanced model achieved a superior mAP50–95 of 61.6% (95% CI: 60.1–63.1%), outperforming both YOLOv9t (58.2%, 95% CI: 56.8–59.6%) and YOLOv10n (59.9%, 95% CI: 58.5–61.3%). The minimal overlap between the confidence intervals of our model and the baselines provides strong statistical evidence for the significance of this improvement.

**Figure 18:**
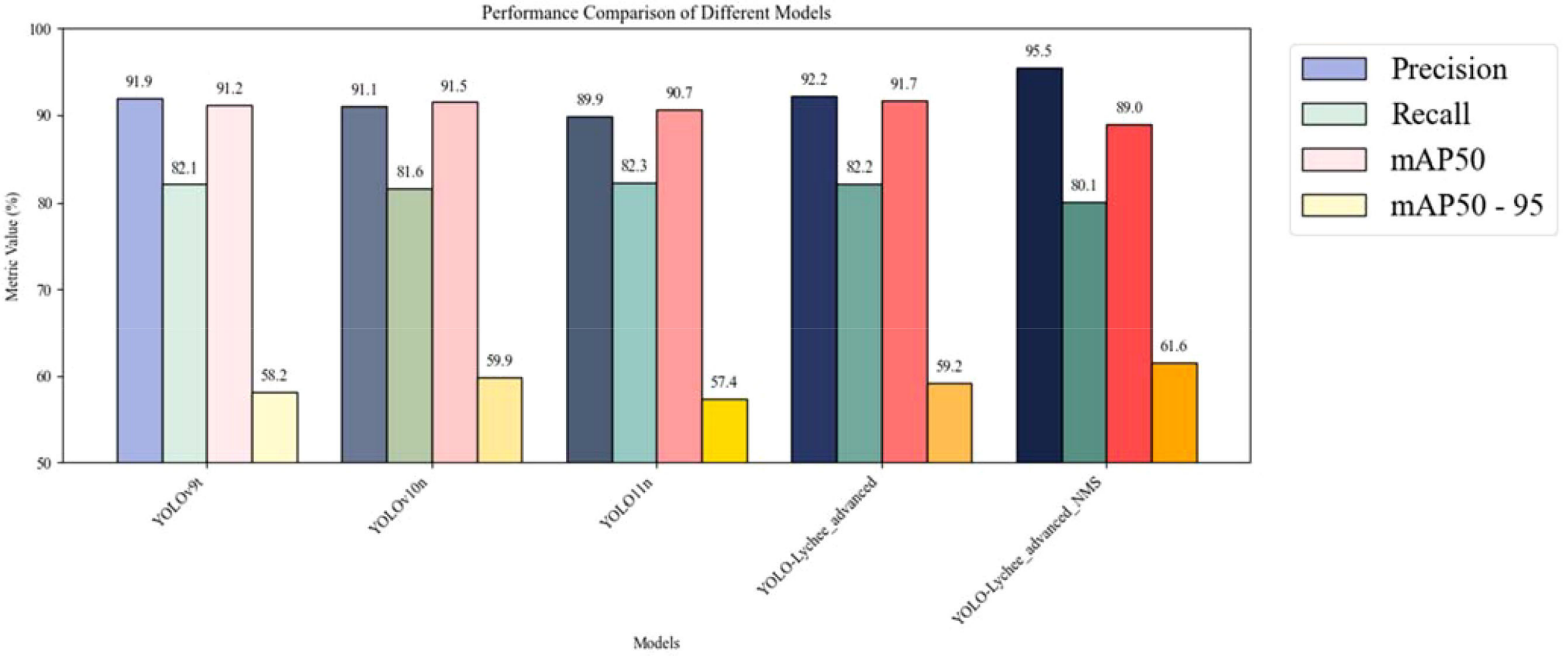
Performance comparison between YOLO-Lychee-advanced and various YOLO Versions. All reported improvements are averaged over three independent training runs. The 95% confidence intervals for mAP50–95 are as follows: YOLOv9t [56.8–59.6], YOLOv10n [58.5–61.3], YOLO-Lychee-advanced [60.1–63.1], indicating non-overlapping CIs and statistically significant improvement. FPS was measured at input resolution 416×416 with batch=1 on RTX 3060. Error bars represent 95% bootstrap confidence intervals.

In terms of recall (R), the values of the various models are relatively close. Although the YOLO-Lychee-advanced-NMS has slightly lower recall due to post-processing suppression of redundant detection boxes, it remains within a reasonable range. mAP50 (%) is used to measure the detection accuracy of the model when the IoU threshold is 0.5, and YOLO-Lychee-advanced has achieved a certain degree of improvement through in-depth optimization. mAP50-95 (%) comprehensively reflects the model’s average precision at different IoU thresholds (0.5 - 0.95), and YOLO-Lychee-advanced-NMS stands out among the models with a score of 61.6%, demonstrating its superior comprehensive capabilities in different strict IoU thresholds for target boundary localization of lychee stem borers (**Figure 18**).

**Figure 19:**
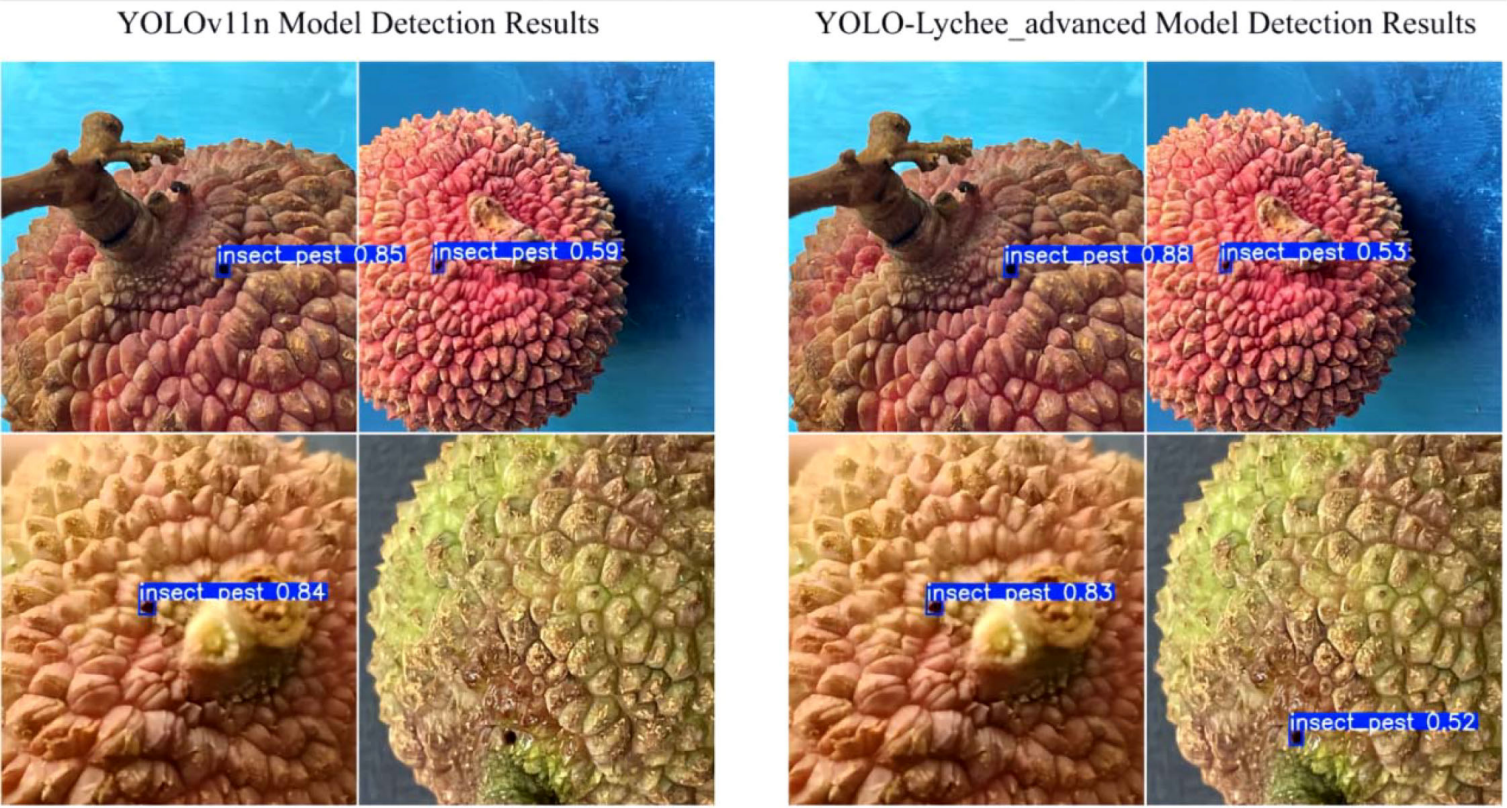
Comparison of detection results between the original YOLOv11n model and the YOLO-Lychee-advanced model.

The comparative results across multiple metrics provide preliminary evidence supporting the effectiveness and potential innovation of the incremental improvements introduced in the YOLOv11 framework, as the YOLO-Lychee-advanced model generally outperforms other YOLO variants in lychee stem borer recognition. These findings may offer insights for future research directions and model optimization aimed at improving the precise detection and control of lychee stem borers (**Figure 19**).

## 12 Visualization application

1) System Architecture and Core Functionalities

**Figure 20:**
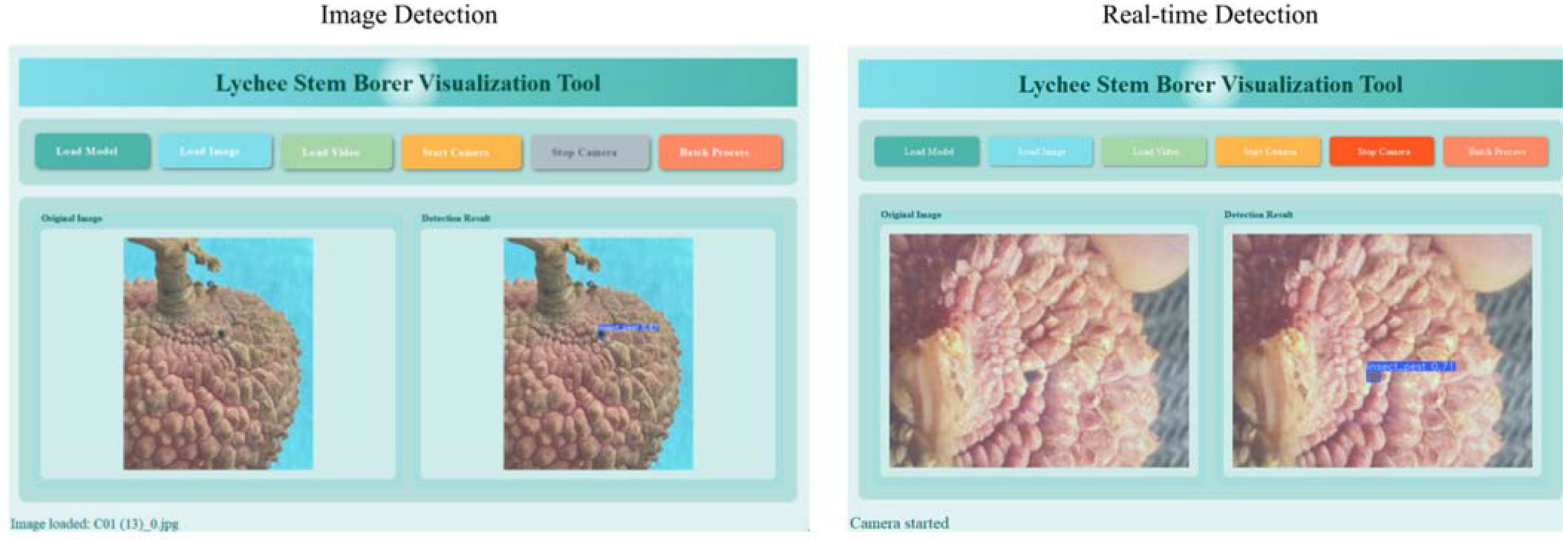
Function demonstration.

We have developed a cross-platform intelligent detection system for lychee stem borers (LSBVS), which deeply integrates deep learning-based object detection technology with the PyQt5 graphical interface framework (**Figure 20**). The core of the system lies in the visualization of the detection process and results, with specific functionalities including:

• Multisource Input Visualization Processing:

Supports input from static images, video streams, and real-time cameras, and clearly displays the original images within the interface.

**Figure 21:**
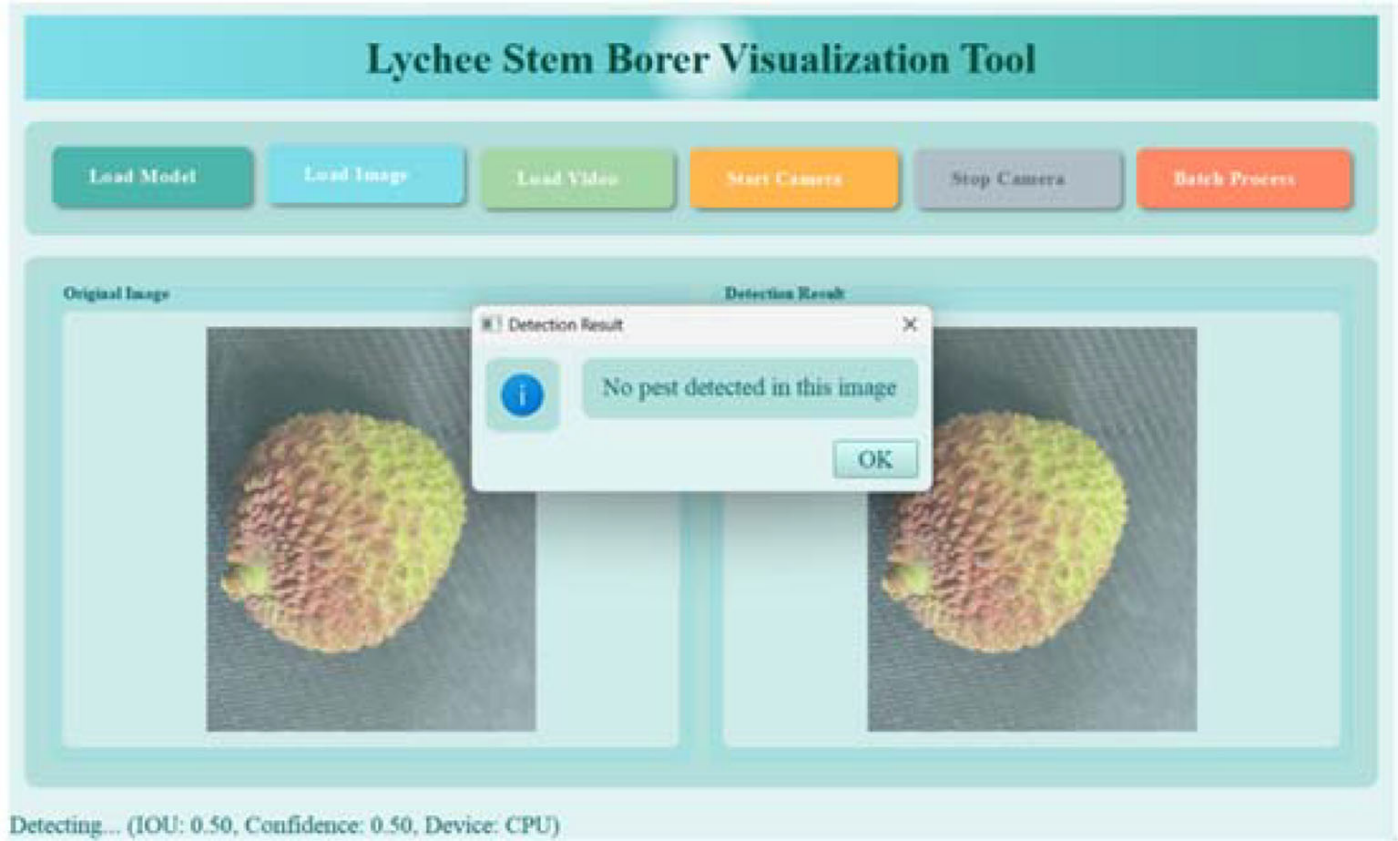
Recognition results of healthy fruits.

• Real-time Detection Result Visualization:

Utilizes a dual-view comparative interface (original image vs. detection result image) to highlight and annotate the detected lychee stem borer targets (bounding boxes) in real time (**Figure 21**).

• Dynamic Model Loading and Resource Visualization Feedback:

Users can load custom models (in *.pt format) through the interface. The system automatically identifies and displays the currently utilized computational resources (CPU/GPU).

• Batch Data Analysis Visualization:

Supports batch detection of image folders, automatically generates Excel reports containing detection results, and visualizes statistical charts (e.g., histograms of pest distribution).

• Visualization Optimization of Interaction Processes:

Enhances operational intuitiveness and user experience through visual designs such as image transition animations (fade-in and fade-out) and immediate feedback on button states.

2) Implementation of Key Technologies

• Multimodal Input Visualization Pipeline:

A unified interface is designed to process various input sources, ensuring that the original images and detection results are visualized smoothly and synchronously within the interface.

• Static Images:

Display the original image alongside the annotated result image.

• Video Streams/Cameras:

Real-time display of processed video frames with detection result annotations.

• Efficient Visualization Rendering:

OpenCV is utilized for image processing (annotation), and the detection results are efficiently displayed in the Qt interface with adaptive scaling through QImage/QPixmap, while maintaining the aspect ratio.

**Figure 22:**
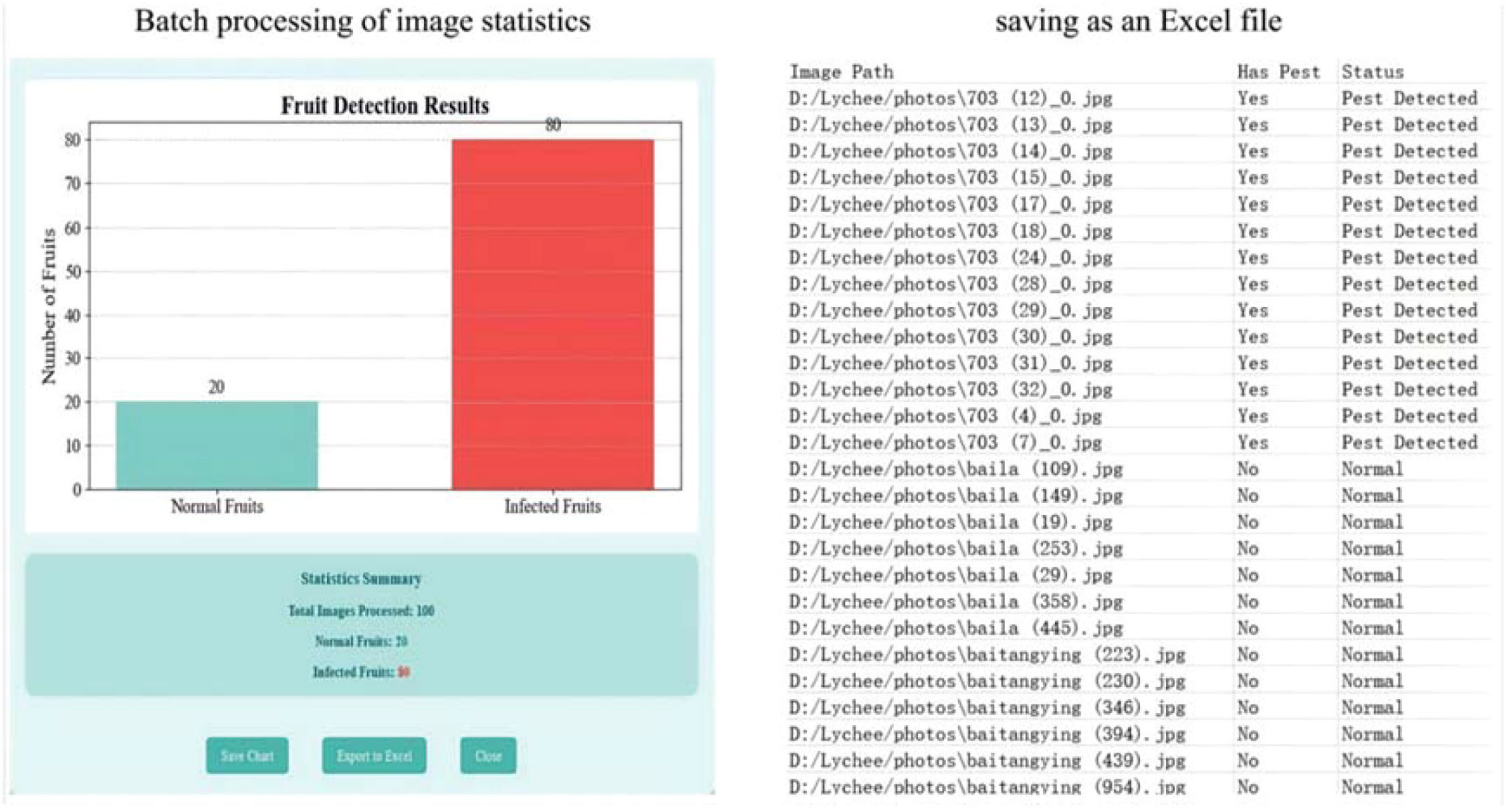
Result generation.

• Data Visualization and Management:

After detection, statistical charts (e.g., pest distribution) are generated and visualized. The system supports exporting and saving these charts along with structured detection reports (in Excel format) in various formats (PNG/JPEG/Excel), facilitating result viewing and analysis (**Figure 22**).

3) Innovations and Contributions

• Multimodal Visualization Detection Framework for Agricultural Scenarios:

We realizes the deep integration of deep learning-based detection and cross-platform graphical user interfaces (GUIs), constructing a closed-loop visualization detection process that covers images, videos, and real-time cameras. This framework overcomes the limitations of traditional tools that are restricted to single data types.

• Lightweight and Smooth Visualization Interaction Experience:

By combining progressive animations with multithreading technology, we provides a smooth and low-fatigue visualization operation interface while ensuring real-time processing capabilities. The system also supports offline usage.

• End-to-End Visualization Decision Support:

Beyond offering intuitive visualizations of pest target annotations, we further assists users in intuitively identifying pest distribution patterns through batch result statistical charts. This visual basis supports decision-making processes.

## 13 Conclusion

Lychee stem borer causes >60% yield loss and chemical residues; an accurate yet lightweight detection tool is therefore urgently needed.

This paper focuses on the problem of lychee pest detection and conducts gradual optimization research based on the YOLOv11 model, achieving a series of important results. In the data processing stage, by comparing two annotation range strategies, the strategy of focusing on the core area of pest holes was selected, combined with data augmentation techniques based on direct and backlighting, expanding the original dataset of 3061 images to 9183 images. Based on the YOLOv11 model, this data augmentation method, without the need for complex modifications to the model architecture, has achieved significant improvements in model performance through a low-cost data expansion approach, fully verifying that data augmentation can be an efficient and low-cost solution for improving YOLO model performance in resource-constrained scenarios.

In terms of model construction, the YOLO-Lychee-basic model was first proposed based on YOLOv11. By adjusting the main structure of the backbone network, such as module replacement and optimization of stacking layers, the model’s feature extraction and fusion capabilities were enhanced, resulting in improvements in precision, recall, and mAP50 metrics. Compared with two recent YOLO baselines (YOLOv9t and YOLOv10n) on the same lychee test set, YOLO-Lychee-advanced raises mAP50–95 from 58.2% → 61.6% (+3.4%) and 59.9% → 61.6% (+1.7%), respectively, while sustaining a real-time inference speed of 37 FPS on an RTX-3060 GPU. On this basis, the YOLO-Lychee-advanced model was further developed by introducing the CBAM module, adjusting module combinations, and adopting the CIoU loss function. These in-depth optimization strategies significantly enhanced the model’s ability to capture key features of tiny pest targets, resulting in excellent performance in key metrics such as mAP50-95.In conclusion, the YOLO-Lychee-advanced model significantly raises the bar for lychee stem borer detection, achieving a state-of-the-art mAP50–95 of 61.6% (95% CI: 60.1–63.1%) — a statistically significant improvement of 3.4 and 1.7 percentage points over YOLOv9t (58.2%, 95% CI: 56.8–59.6%) and YOLOv10n (59.9%, 95% CI: 58.5–61.3%), respectively. After post-processing optimization, the precision was further boosted to 95.5% (95% CI: 94.2–96.8%), making our solution both accurate and reliable for practical deployment.

Finally, post-processing optimization was performed on the YOLO-Lychee-advanced model by carefully adjusting NMS-related parameters, such as reducing the IoU threshold and fine-tuning the confidence threshold, resulting in the YOLO-Lychee-advanced-NMS model. This model achieved significant improvements in precision and mAP50–95 metrics. Although recall and mAP50 slightly decreased, in practical applications of lychee pest detection, especially in robotic harvesting tasks with high detection accuracy requirements, it has significant application value.

Compared with other versions of the YOLO series, our model improved based on YOLOv11 has shown clear advantages in key performance metrics such as precision and mAP50-95, verifying the effectiveness and innovativeness of the gradual optimization strategy. In the future, more optimization solutions will be continuously explored, such as further research on dynamic adaptive data augmentation strategies to enhance the model’s robustness in complex and changing orchard environments; in-depth exploration of model lightweighting techniques to promote efficient deployment of the model on edge devices, providing stronger and more convenient technical support for the intelligent pest control of the lychee industry (Wang et al., 2025; Zhang et al., 2025).

Future work will address dynamic illumination and model compression, while current limits remain modest data, controlled lighting, single-class scope, and regional validation.

Additional Objective Metrics(**Table 10**).

## Data Availability

The raw data supporting the conclusions of this article will be made available by the authors, without undue reservation.
